# Person-centred environments and nurses**’** turnover intention: examining their relationship using structural equation modelling

**DOI:** 10.3389/frhs.2026.1816479

**Published:** 2026-07-15

**Authors:** Martin Wallner, Birgit Schönfelder, Johannes Michael Bergmann, Thomas Falkenstein, Ursula Gössl-Lurf, Oliver Radinger, Hanna Mayer

**Affiliations:** 1 Department of Nursing Science with Focus on Person-centred Care Research, Faculty of Health Sciences, Karl Landsteiner University, Krems, Austria; 2 Faculty of Health, University of Applied Sciences Wiener Neustadt, Wiener Neustadt, Austria; 3 Münster Department of Health, FH Münster University of Applied Sciences, Münster, Germany; 4 Vienna Health Care Group, Executive Board Department, Department of Human Resources Development & Education, Vienna, Austria

**Keywords:** coolout, intention to leave, job satisfaction, moral distress, person-centred care, practice environment, structural equation modelling, turnover intention

## Abstract

**Background:**

A supportive practice environment is essential for nurse retention and to enable nurses to provide effective person-centred care. When nurses are prevented from acting according to their professional values, they may experience moral distress, potentially leading to disengagement and increased turnover intention. Although the impact of the practice environment on nurse outcomes is well documented, the pathways linking person-centred aspects of the practice environment to turnover intention remain underexplored. This study aimed to test a theoretical model linking these factors to inform targeted retention efforts.

**Methods:**

We conducted a cross-sectional study of nurses (*n* = 573) across several acute-care hospitals and nursing homes within a large healthcare organisation. Validated instruments were used to assess key constructs. Model A posited that nurses’ prerequisites, the practice environment, and person-centred processes predict the perceived person-centred climate. Model B examined whether person-centred climate influences turnover intention, mediated by moral distress, coolout, and job satisfaction. Structural equation modelling was performed using the lavaan package in *R*.

**Results:**

Both models showed acceptable fit following theoretically justified modifications (Model A: CFI = 0.930, RMSEA = 0.060; Model B: CFI = 0.926, RMSEA = 0.047). In Model A, predictors explained 51.8% of the variance in person-centred climate. The practice environment emerged as the sole significant predictor of a person-centred climate (*β* = 0.74, *p* < 0.001) and mediator (*β* = 0.50, *p* < 0.001) between nurses’ prerequisites and person-centred climate. In Model B, predictors explained 57.3% of the variance in turnover intention. Person-centred climate was indirectly associated with lower turnover intention via job satisfaction (*β* = −0.468, *p* < 0.001) and, to a lesser extent, via both job satisfaction and coolout (*β* = −0.124, *p* < 0.001). The indirect path through moral distress was negligible and non-significant (*β* = 0.043, *p* = 0.067).

**Conclusions:**

Findings can be used to inform retention strategies at both the meso and micro levels, including the development of monitoring tools for turnover intention and measures to support practice development for teams and individual practitioners. Future research should further explore mediating mechanisms to inform retention efforts.

## 1 Introduction

The shortage of qualified nursing staff is a prevailing challenge in healthcare globally, pertaining not only to the availability of nursing staff but also to the ability to retain qualified nurses (1). While Austria has a relatively high nurse density (2) of 10.6 nurses per 1,000 population (3), it is not immune to workforce pressures driven by demographic changes and working conditions (4, 5). Projections indicate that by the year 2050, an estimated 70.000 nursing positions will need to be replaced or newly filled to maintain current levels of care (6), corresponding to an additional demand for nurses of roughly 58%. These workforce pressures can result in overworked and dissatisfied staff, reduced quality of care, and consequently negative effects on patient outcomes (7).

The situation is exacerbated by high staff turnover rates and a high prevalence of turnover intention, i.e., the desire of nurses to leave their current workplace (organisational turnover) or the nursing profession (professional turnover). Organisational turnover intention reflects a more immediate dissatisfaction with unfavourable working conditions, whereas intention to leave the profession reflects a broader erosion of professional commitment and identity (8, 9). Turnover occurs particularly in work environments with high stress levels, emotional exhaustion, impaired integrity, burnout, dissatisfaction, and lack of support (10–13). Nurses’ intention to leave is considered the strongest predictor of actual turnover (14, 15). Determinants include an interplay of individual, interpersonal, organisational and macro level factors (16). Global meta-analytic studies suggest a nurse turnover rate of 16% (17) and a prevalence of turnover *intention* of 38% (18), with high variation by country, specialty and work environment. For Europe, one study found a nursing turnover intention prevalence of 9% across ten European countries (19). Another study across six European countries observed a rate of 33% (20). Recent studies from Austria are even more alarming, with estimates of turnover intention in nurses ranging from 64% (5) to 75% (4). Nevertheless, there is important nuance to turnover (intention), as it may be voluntary or involuntary, avoidable or unavoidable, and driven by internal or external reasons, which complicates comparisons across studies (14).

One of the strategies to address these workforce pressures is to strengthen nurse retention. One key approach is developing and maintaining supportive, person-centred practice environments and supporting the well-being of nurses (7). This includes aspects such as teamwork, support from leaders, and power sharing (21). Leaders play an essential role in developing the practice environment and thus ensure the necessary conditions for providing high-quality nursing care (21). The relationship between a supportive practice environment and various nursing outcomes has been demonstrated in numerous studies. These include nurses’ wellbeing, job satisfaction, as well as the intention to stay in the current workplace (14, 22–25). However, much of this literature conceptualises the practice environment broadly, with limited consideration of specific aspects grounded in person-centred care theory.

A person-centred climate represents specifically psychosocial and interpersonal dimensions of the practice environment, reflecting the attitudes and actions of individuals and the underlying philosophy of care within organisations (26). A supportive, person-centred culture is expected to contribute to job satisfaction among nursing staff (27, 28) and leads to improved care quality and lower staff turnover (29). By contrast, studies show that the perceived quality of care decreases when nurses are exposed to morally distressing situations (30, 31). Moral distress occurs when nurses are prevented from acting in accordance with their moral and ethical principles (32). This can negatively affect job satisfaction and increase staff turnover (33–38). Over time, moral distress may lead to moral desensitisation, emotional exhaustion, and detachment, a phenomenon conceptualised as cool down or coolout (39–41). Coolout represents a coping strategy that enables nurses to maintain their work performance, albeit at the expense of emotionally engaged patient care (39) as they establish personal moral and ethical boundaries that, once exceeded, may ultimately lead them to leave their institution or the profession (42). While these factors have been examined individually, prior research has not integrated them into a comprehensive explanatory framework. There is a lack of evidence on how person-centred aspects of the practice environment may mitigate moral distress and emotional disengagement, thereby indirectly influencing turnover intention.

This gap is particularly relevant for the Austrian context, where high turnover intention rates suggest that existing models may not fully capture the dynamics at play. Since practice development efforts to foster person-centred cultures require substantial long-term organisational commitment (43), it is essential to better understand the underlying factors to justify and guide such investment. Moreover, monitoring workforce developments and generating context-specific evidence is considered a key policy priority, given substantial variation in staffing and retention factors across countries (7). While existing literature suggests that person-centred workplace cultures may protect against moral distress and emotional disengagement among nursing staff, therefore supporting their intention to remain in their current workplace, evidence clarifying these relationships for the Austrian context is limited.

This study therefore aimed to develop and test a theoretical model that explains the relationships between the practice environment and nurses’ turnover intention as well as mediating factors, including person-centred climate, moral distress, coolout, and job satisfaction. By examining these relationships for the Austrian context, we seek to provide a more nuanced understanding of the factors associated with nurse retention and to inform targeted strategies for improving workforce stability.

## 2 Theoretical background and hypotheses

### 2.1 Person-centred environments

A person-centred climate relates to the overall psychosocial atmosphere experienced within the care environment. Specifically, it refers to an environment in which individuals feel welcome, safe, and at home in their daily experiences. It emerges from the joint contribution of the setting and staff, who together create conditions that protect personhood and allow individuals to be persons, rather than merely patients or workers (26, 44, 45).

Person-centred care describes an approach to nursing practice that places the patient as a person at the centre of decision making. Person-centred care has a long association with the nursing profession, as it involves caring for people as individuals, respecting their rights as persons, building mutual trust and understanding, and developing a therapeutic relationship (29). While the focus of person-centred care has traditionally been patients, this has gradually expanded, with increasing attention being given to all individuals involved, including staff. Moreover, nurses must be enabled to work in a person-centred way and to deliver effective person-centred care (21, 46). In this understanding, person-centredness is defined as “[…] an approach to practice established through the formation and fostering of healthful relationships between all care providers, service users and others significant to them in their lives. It is underpinned by values of respect for persons, individual right to self-determination, mutual respect and understanding. It is enabled by cultures of empowerment that foster continuous approaches to practice development” (47).

One of the most elaborate approaches to person-centredness is articulated in the Person-centred Nursing Framework by McCance and McCormack (29). While this framework is specifically designed for nursing practice and is grounded in the nursing metaparadigm (29), an alternative version exists that encompasses all healthcare professionals and addresses factors such as workforce developments in the macro context (46). Nevertheless, both frameworks share key theoretical constructs at the meso and micro level. According to these frameworks, the aim is to create a positive care experience for everyone involved, including patients, relatives, and nursing staff. Achieving this goal depends on the characteristics of the nurses (prerequisites) and on various aspects of the care environment. Together, these enable nurses to deliver person-centred care (21). A person-centred approach in nursing is expected to not only increase patient satisfaction but also job satisfaction among nurses (24). Nurses who perceive their working environment as person-centred are more satisfied and less likely to consider leaving the profession (11, 13).

### 2.2 Job satisfaction

Job satisfaction refers to the extent to which people like (satisfaction) or dislike (dissatisfaction) their work (48). Factors determining satisfaction include a positive work environment, recognition by leaders, social relationships, opportunities for advancement, and taking on tasks that align with personal ethical values (11, 13). If employees have little influence over their work environment, this negatively influences job satisfaction. High stress combined with reduced job satisfaction is associated with high turnover rates (11, 13). Conversely, when ethical expectations are supported through person-centred care and collaboration with patients is encouraged, job satisfaction increases (24).

### 2.3 Moral distress

Moral distress is defined as a psychological imbalance and a negative emotional state that occurs when a person makes a moral judgement but does not or cannot carry out the resulting moral action (37). Moral action represents a fundamental attitude to which members of the nursing profession are committed (49). The nursing profession is perceived as meaningful and valuable when nurses can act in accordance with their values and principles and feel that patients are being cared for in the right way (35, 37, 50). Moral and ethical problems may arise from moral uncertainty and dilemmas (50), but also from role conflicts and discrepancies between personal values and the value system of an organisation (51). For example, situations in interprofessional collaboration where nurses feel that actions are not taken in the patient's best interest, feeling helpless when pain management is inadequate in their view (38), or the objectification of patients (37). A constantly changing healthcare system and high workloads caused by complex and often unfavourable conditions also complicate everyday nursing practice (52).

Moral distress is associated with post-traumatic stress, feelings of guilt, relationship fatigue, burnout, as well as moral injury and moral failure, particularly when the distress is prolonged (30). Feelings of frustration, anger and fear regarding the moral beliefs of individuals in the organisation may occur, as well as physical symptoms such as headaches, insomnia, and feelings of shame and guilt (37, 38). As a consequence, feelings of uselessness and overwhelm may occur, leading to distancing and reduced empathy towards patients (37, 38, 50), affecting patient well-being and quality of care (30, 31), leading to increased job dissatisfaction and turnover (34–37). However, moral distress can also be understood as an expression of moral sensitivity, which is considered indispensable in this field (30). The aim is therefore not to avoid moral distress in every clinical situation, as it directs attention toward professional values and norms and helps preserve moral integrity (30, 31).

### 2.4 Coolout/cool down

The term coolout describes a progressive process of moral desensitisation. Because of the conflict between normative expectations and the actual reality of daily work, nurses develop strategies that can appear as emotional coldness in everyday nursing practice (42). A major source of coolout is a perceived discrepancy between patient-oriented care and demands related to system rationality in daily work. A patient-centred orientation of care means that nursing focuses on the needs of the individual. Patients and nurses are equal partners working together to achieve the intended goals. In contrast, system rationality demands efficient and standardised procedures, where individuality can only be considered to a limited extent. Depending on workload and available time, communication is reduced to what is strictly necessary, followed by cutbacks in physical care, with little time left for human attention. When the required standard of care can no longer be met, this can result in emotional coldness in nurses as a reaction to structural conditions (53).

Büssing and colleagues (39, 40) describe a similar phenomenon using the term cool down. Cool down is understood as a coping strategy that manifests as emotional exhaustion and distancing to maintain one's own functionality at work. Nurses are frequently confronted with high staff turnover, heavy workloads, staff shortages, shift work, night work, and a high number of complex and multimorbid patients. In addition, they face strong moral and emotional demands as well as a strong sense of responsibility for their work and their patients. This can cause nurses to feel emotionally exhausted, less satisfied with their work or life situation, or to consider leaving their job. To cope with this situation, nurses develop strategies that allow them to stay in their jobs and to continue functioning. This can include a conscious decision to withdraw emotionally (39, 40). Emotional exhaustion can manifest as an impersonal, detached and sometimes cynical attitude towards patients. As a result, care is increasingly experienced as a duty and the quality of care decreases. If suitable coping mechanisms are lacking, emotional exhaustion can lead to an inability to work (39).

### 2.5 Proposed theoretical model and hypotheses

Our theoretical model posits that turnover intention of nurses develops gradually over time, resulting from an imbalance between supportive and impeding factors related to nurse well-being. Supportive factors include a positive, person-centred work environment that enables nurses to work in a person-centred way and in accordance with their professional ethos. This increases job satisfaction, compensates for existing moral distress and prevents emotional withdrawal, which in turn promotes nurses’ intention to stay in their current workplace and in the nursing profession. Conversely, when the work environment is deficient and nurses are unable to act in line with professional principles, moral distress is expected to emerge first and job satisfaction to decline, reflecting an early imbalance between demands and professional standards. Sustained moral distress and reduced job satisfaction may then trigger emotional withdrawal (cooling down/coolout) as a maladaptive coping response. This withdrawal is posited to precede and contribute to nurses’ intention to leave the current workplace or the profession.

The model comprises three mechanisms. First, organisational and contextual factors (PRE, ENV, PRO) shape the psychosocial climate (PCQ), with prerequisites (PRE) of nurses and a favourable practice environment (ENV) further facilitating person-centred processes (PRO). Second, PCQ influences key work-related outcomes, being associated with higher job satisfaction (JS) and lower moral distress (MD). Third, downstream outcomes are conceptualised as a sequential process: an unfavourable climate initially increases moral distress, which is followed by coolout/cool down (CO) as a maladaptive coping response, ultimately leading to higher intention to leave (IL). In parallel, job satisfaction exerts a protective effect by reducing both coolout and intention to leave. Indirect effects are specified and tested as specific and serial mediation pathways.

**Figure 1:**
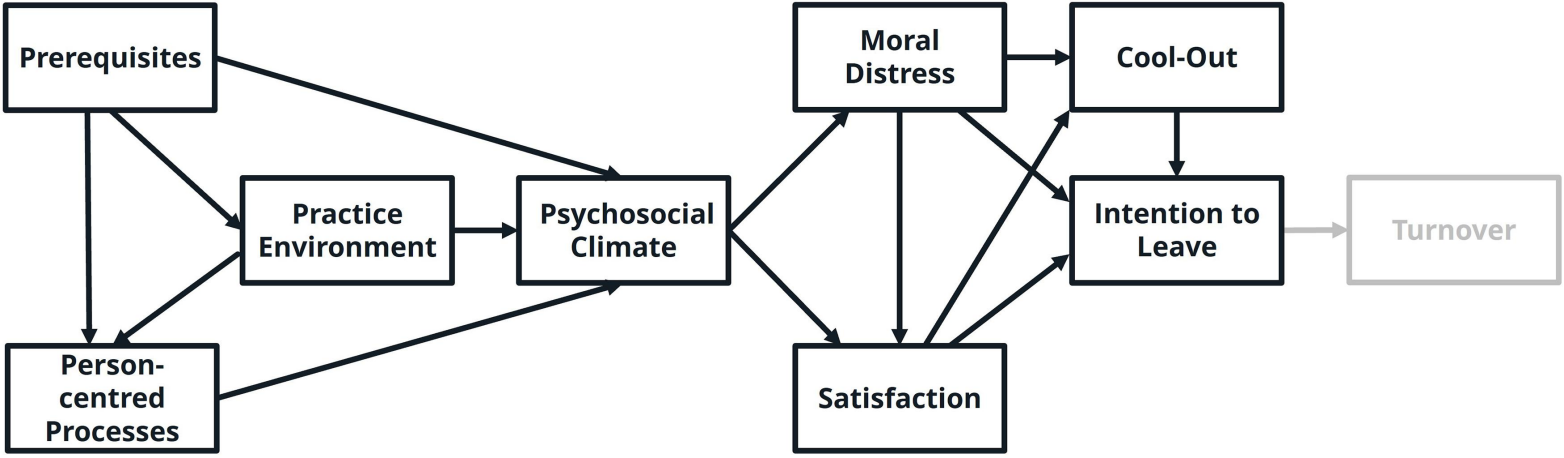
Proposed theoretical model illustrating the hypothesised relationships among constructs.

Building on the theoretical model presented in Figure 1, we specify two structural equation model-based path models, one linking personal, organisational and process-related determinants to the perceived psychosocial climate (PCQ), the other linking a psychosocial climate to work-related core outcomes (moral distress and job satisfaction) and downstream outcomes resulting in intention to leave. To avoid overloading the analysis with numerous individual hypotheses, we group the directed relationships into three blocks (H1-H3: upstream determinants, mediator outcomes, and downstream outcomes). In addition, we define a limited number of *a priori* specified indirect associations that capture the model's core mediation assumptions (H4).

**H1 (Upstream determinants of PCQ)**:

- Prerequisites (PRE) shape the practice environment (ENV) and person-centred processes (PRO) (PRE→ENV, PRE→PRO, all positive);
- PRE and ENV additionally support PRO (PRE→PRO, ENV→PRO, all positive);
- Collectively, PRE, ENV and PRO contribute to a more positive psychosocial climate (PCQ) (PRE/ENV/PRO→PCQ; all positive).

**H2 (PCQ → core outcomes):**

- A more positive PCQ increases job satisfaction (JS) and reduces moral distress (MD) (PCQ→JS positive; PCQ→MD negative).

**H3 (Downstream cascade to IL):**

- MD decreases JS and increases CO and IL (MD→JS negative; MD→CO positive; MD→IL positive)
- JS decreases CO and IL (JS→CO negative; JS→IL negative)
- CO increases IL (CO→IL positive).

**H4 (Mediation/serial mediation)**: We test priori specified indirect effects within predefined pathways:

- (a) PRE→PCQ via ENV, and PRE→PRO via ENV;
- (b) PCQ→IL via JS, CO, and MD

As no indirect paths from PRE, ENV, or PRO to MD or JS are specified, the model implies that, after adjustment for PCQ, these antecedents should not exhibit any additional association with MD or JS.

## 3 Materials and methods

### 3.1 Design and sample

A cross-sectional design using an online survey was utilised in this study. The objective was to develop and test a theoretical model examining the relationship between person-centred environments and turnover intention.

The study was carried out at 10 of the 19 sites within a large healthcare organisation in Austria, selected to be representative of the overall structure and distribution of settings. Settings included acute care hospitals, nursing homes, and psychiatric care facilities. The target group for the study comprised individuals employed in a nursing role at the time of data collection, including registered nurses (RNs) as well as first and second level nursing assistants, with one and two years of training, respectively. The number of eligible nursing staff was *N* = 5,191 out of a total workforce of 11,751 nurses.

Nurses were recruited using a convenience sample. Given the complexity of the model (multiple latent constructs, as well as multiple direct effects and serial indirect paths), no precise *a priori* sample size calculation was conducted, as this would have required robust assumptions regarding effect sizes and variance-covariance parameters that could not be sufficiently justified in the present context. Instead, a pragmatic target (≥400 cases per sample) was adopted as a lower-bound guideline. *post hoc* (“observed”) power was not reported, as it largely reflects the *p*-value retrospectively and therefore provides no additional information for the interpretation of non-significant findings (54).

### 3.2 Instruments

Sociodemographic data were collected, including gender, age, professional group, qualifications, professional experience, educational level, specialty, and type of employment.

Validated German-language versions of instruments were used to assess key constructs. Consent to use the instruments was obtained from the respective authors.

The Person-centred Practice Inventory–Staff (PCPI-S) was used to measure the staff perception of the person-centredness of the practice environment. Originally developed by Slater and colleagues (55), the PCPI-S is available in several languages, including an Austrian-German version, which was translated and psychometrically tested in long-term care by Weis and colleagues (56). The instrument consists of 17 dimensions with 59 items, formulated as statements covering three constructs: prerequisites, practice environment, and person-centred processes. Nursing staff are asked to rate statements on a five-point Likert-type scale (1 = “strongly disagree” to 5 = “strongly agree”) (55, 56).

The perception of the psychosocial climate was measured using the Person-Centred Climate Questionnaire – Staff (PCQ-S). The PCQ-S is a widely used and tested scale for evaluating person-centred practice environments. It is based on theoretical considerations regarding how a supportive environment can protect personhood. The original instruments for staff, residents in long-term care, and their relatives were developed in Sweden and translated into numerous languages (57), including German (58, 59). The staff version comprises 14 statements rated on a six-point Likert-type scale (1 = “No, I disagree completely” to 6 = “Yes, I agree completely”). The instrument is divided into the subscales Safety, Everydayness, and Community (57–59).

Moral distress was measured using the Moral Distress Scale (MDS). The German version was translated and adapted by Kleinknecht-Dolf and colleagues (32) based on the American version by Hamric and Blackhall (60) for the hospital setting. The current version consists of 13 items. The first item assesses the frequency of adhering to ethical principles in nursing decision-making, rated on a five-point verbal rating scale (1 = “never” to 5 = “several times a week”). The remaining 12 items address experiences in clinical practice that may trigger moral distress. Respondents are asked how often they encounter such situations (on a five-point scale from 1 = “never” to 5 = “several times a week”) and to rate the degree to which their moral integrity is affected on a second verbal five-point scale (0 = “none” to 4 = “very high”) for each item.

The Cool Down Index (CDI) was used to measure emotional burden and withdrawal in nurses. The CDI, originally developed in German language by Büssing and colleagues (39, 40), assesses the perception of emotional exhaustion in nurses and the resulting emotional withdrawal as a coping strategy. The CDI consists of nine items with two subscales (emotional distancing, emotional withdrawal). While initially, both frequency and intensity of emotional withdrawal were assessed, more recent publications focus on the frequency of the corresponding feelings and reactions when interacting with patients, using a six-point scale (1 = “a few times a year or less” to 6 = “every day”) (61).

Two subscales of the Nursing Context Index (NCI) were used to assess turnover intention and job satisfaction. Originally developed by Slater and colleagues (24), the NCI was translated into German in 2018 by researchers at the Department of Nursing Science, University of Vienna (unpublished). Job satisfaction entails personal satisfaction and professional satisfaction and was measured using an adapted 5-point scale (1 = “strongly dissatisfied” to 5 = “strongly satisfied”) (24). Intention to leave was measured using the 2 items of the NCI subscale, and one item was added to reflect intention to leave the nursing profession (1 = “strongly disagree” to 5 “strongly agree”).

### 3.3 Data collection

Data collection was carried out using a secure online survey platform (REDCap), which was hosted on the server of [Karl Landsteiner University]. The survey URL was distributed to the target group by the responsible person at [Vienna Health Care Group]. The survey took approximately 30–40 min to complete and was conducted between 24 October and 2 December 2024.

### 3.4 Data analysis

Data preparation was carried out using the Statistical Package for the Social Sciences 29 (62) and Microsoft Excel (63). Data analysis was conducted using *R* (64) in jamovi 2.6.26 (65), including the module SEMLj (66), as well as *R* in RStudio 2024.09.0 + 375 (67), including the packages lavaan version 0.6-19 (68), lavaangui version 0.2.4 (69), semTools version 0.5-8 (70) and psych version 2.5.3 (71).

Descriptive statistics were used to analyse sample characteristics. Structural equation modelling (SEM) was used to evaluate model fit to the data and to analyse relationships of the hypothesised theoretical model.

SEM refers to a set of statistical techniques used to estimate the strength and direction of hypothesised structural relationships. Data from observed (manifest) variables are treated as approximations of hypothetical (latent) constructs. By using manifest variables as indicators for latent constructs, it is possible to estimate relationships between the latent constructs. SEM can be applied in both explorative and confirmative research (72). SEM is typically implemented in two steps. First, a measurement model is specified and tested with confirmatory factor analysis to link observed variables to latent constructs. After evaluating and adjusting the measurement model, a structural model is specified to define relationships among latent variables, representing the theoretical framework. If the structural model fits well, the parameter estimates, including effect sizes, can be interpreted (73). SEM reporting followed the APA Journal Article Reporting Standards for Quantitative Research (JARS–Quant) (74).

Mardia's tests of multivariate skewness and kurtosis were significant (both *p* < 0.001), indicating violation of multivariate normality. Accordingly, the Yuan-Bentler correction (MLR), a robust extension of the maximum likelihood method, was used for model estimation (73). MLR was selected as the primary estimator due to the complexity of the structural model and because ordinal estimators (WLSMV/DWLS) did not converge to stable, admissible solutions.

To optimise model convergence, parameter reduction of the PCPI-S was conducted using facet-representative item parcelling (75), resulting in 17 observed variables instead of 59. Parcelling was applied only to previously validated instruments with an established theoretical and factorial structure. Parcels were created in line with the predefined subscale structure and not as a data-driven strategy to improve model fit. Since the aim of the SEM was to examine structural associations between established constructs rather than to revalidate the measurement models at item level, this approach was considered appropriate for reducing model complexity and improving estimation stability.

The significance level for inferential statistics was set at *α* = 0.05. Standardised factor loadings greater than 0.5 were considered acceptable (76). Standardised path coefficients (*β*) greater than |0.2| were regarded as meaningful [Chin, 1998, cited in (77)]. The models were refined in an iterative process using modification indices, allowing only theoretically justified modifications (details in results section and supplementary material). Criteria used to assess model fit are outlined in Table 1 [76, 88].

**Table 1:** Criteria to assess model fit (76, 88).

Fit index	*N* > 250, m ≥ 30
*χ*2/degrees of freedom (df)	*χ*2/df ≤ 3
Root mean square error of approximation (RMSEA)	< 0.07
Comparative fit index (CFI)	≥ 0.92
Tucker Lewis Index (TLI)	≥ 0.92
Standardised root mean square residual (SRMR)	≤ 0.08

N, sample size; m, number of observed variables in the model.

Prior to analysis, data were screened using predefined criteria (e.g., >10% missing responses; frequently repeated responses, defined as cases in which identical responses were provided for all items across more than one instrument) which reduced the extent of missingness and implausible response patterns. Little's MCAR test was conducted for the overall SEM variable set and separately for each instrument; results were mixed across instruments. Given that MCAR is not required for likelihood-based handling of missing data, all SEMs were estimated using FIML (with robust standard errors where applicable), which yields unbiased and efficient estimates.

### 3.5 Ethical considerations

The study was approved by [Vienna Health Care Group] as well as an employee representative. Data collection was conducted anonymously, and sociodemographic data were collected using categories. Participation was voluntary. Study information was provided by the responsible persons at the participating sites. Additionally, information on the study's aims and procedures was offered to participants at the start of the online survey. Participants explicitly consented before beginning the survey by agreeing to a corresponding statement. Decisions not to participate could not be traced back to individual nursing staff. Researchers had no access to personal data. Research data files were stored exclusively on the university server and were accessible only to members of the research team. In Austria, ethical board approval is not required for non-interventional studies, in accordance with national research ethic regulations.

## 4 Results

### 4.1 Participant characteristics

A total of 1,038 entries were recorded in the online survey tool. After removing empty cases (*n* = 148), cases with more than 10% missing responses (*n* = 266), and frequently repeated responses (*n* = 51), the final sample for model testing comprised *n* = 573 cases, corresponding to a response rate of 11% (573/5159).

Most participants (81.8%) worked in hospitals. More than three-quarters (78.8%) identified as female and one-fifth (21.0%) as male. One-fourth of participants reported their age as 40–49 years (27.4%), one-third as 50–59 years (36.0%), and one-fifth aged 30–39 years (20.6%). The majority of participants were registered nurses (88.3%), while first level nursing assistants (4.9%) and second level nursing assistants (5.2%) combined accounted for approximately one-tenth of the sample (Table 2).

**Table 2:** Participant characteristics.

Variable (n)	Category	Sample (*N* = 573)		National workforce* (*N* = 179,041)
		*n*	%	%
Care setting (*n* = 573)	Acute care hospital	469	81.8	–
	Nursing home	32	5.6	–
	Psychiatric care facility	72	12.6	–
Gender (*n* = 567)	Female	446	78.8	85
	Male	119	21.0	15
	Non-binary	2	0.4	–
Age in years (*n* = 573)	≤ 19	0	0.0	–
	20–29	64	11.2	14
	30–39	118	20.6	25
	40–49	157	27.4	22
	50–59	206	36.0	26
	≥ 60	28	4.9	9
Professional group (*n* = 573)	Nurse assistant (level 1)	28	4.9	33
	Nurse assistant (level 2)	30	5.2	5
	Registered Nurse	506	88.3	62
	Other	9	1.6	–
Qualification (*n* = 503)	Nursing school	445	88.5	80
	University of Applied Sciences	56	11.1	7
	Combined training	2	0.4	–
Education (*n* = 571)	Compulsory school	101	17.7	–
	Vocational school	99	17.3	–
	Intermediary vocational school	60	10.5	–
	Higher vocational school	51	8.9	–
	Grammar school	136	23.8	–
	University (of Applied Sciences)	124	21.7	–
Specialty (*n* = 564)	Yes	303	53.7	–
	No	261	46.3	–
Job experience in years (*n* = 572)	< 1	10	1.7	–
	1–5	74	12.9	–
	6–10	55	9.6	–
	> 10	433	75.7	–
Employment (*n* = 572)	Full time	413	72.2	–
	Part time	159	27.8	–

N/n, Sample sizes; *Workforce data from the Austrian Registry of Healthcare Professions (78); (–) no data.

Sample characteristics were compared with the Austrian Healthcare Professions Register (78), which is mandatory for selected health professionals, including nurses. The sample approximately reflects the population of nurses in terms of gender, age, and nursing qualification. Differences exist in the distribution of professional groups, with RNs being overrepresented in this study.

Participants reported high levels of both prerequisites and person-centred processes, while the practice environment was rated comparatively lower. The person-centred climate was also perceived as moderate to high, although the climate of everydayness received moderate scores. In contrast, moral distress and cool down were reported at moderate to low levels. Job satisfaction was moderate to high, with personal satisfaction exceeding professional satisfaction. Descriptive statistics are presented in Table 3.

**Table 3:** Descriptive statistics of the measured constructs.

Construct	Mean	SD	Skewness	Kurtosis
Prerequisites	4.22	0.514	−0.807	1.3079
Practice environment	3.78	0.645	−0.435	−0.1102
Person-centred processes	4.27	0.540	−0.533	−0.1627
Person-centred climate	4.21	0.810	−0.355	−0.0389
Climate of safety	4.45	0.887	−0.525	0.0753
Climate of everydayness	3.61	1.006	−0.210	−0.3703
Climate of community	4.55	0.912	−0.533	0.3296
Moral distress	2.31	0.869	0.506	−0.4810
Job satisfaction	3.52	0.741	−0.299	−0.1844
Personal satisfaction	3.75	0.790	−0.538	0.1891
Professional satisfaction	3.29	0.835	−0.238	−0.2859
Cool down index	2.29	1.177	0.975	0.2939
Emotional withdrawal	2.18	1.190	1.160	0.6901
Emotional distancing	2.40	1.386	0.850	−0.2551
Intention to leave	2.25	1.219	0.662	−0.7046

SD, standard deviation.

### 4.2 Structural equation modelling

#### 4.2.1 Latent correlations and discriminant validity

Examination of latent construct correlations indicated high correlations (*r* > 0.8) between prerequisites (PRE) and person-centred processes (PRO) as well as person-centred climate (PCQ) and job satisfaction (JS), suggesting substantial conceptual overlap in this sample. The strong positive correlation (*r* = 0.815) between PRE and PRO is consistent with the Person-Centred Practice Framework (46), which posits that practitioners’ professional attributes and values underpin the enactment of person-centred processes during care interactions. The latent association between PCQ and JS was consistently high across model specifications, ranging from *r* = 0.832 in the initial measurement model to *r* = 0.863 in the structural model. However, both constructs were retained based on their theoretical distinctiveness and prior empirical support in the literature. The full correlation matrix was inspected for latent construct correlations (Supplementary Material). No broad pattern of severe collinearity was identified.

In addition to latent correlations, discriminant validity among the JS and PCQ constructs was assessed in the respecified measurement model using the heterotrait-monotrait ratio (HTMT) (79), calculated with the htmt() function in the semTools package (70). Due to the higher-order structure of the measurement model, HTMT was evaluated at the first-order construct level. This approach was chosen because HTMT is based on correlations among indicators directly assigned to their respective latent constructs. HTMT values between the JS dimensions (PERSSAT, PROFSAT) and the PCQ dimensions (SAFE, EVRY, COMM) ranged from 0.401 to 0.836. The highest HTMT value was observed between “a climate of safety” (SAFE) and “professional satisfaction” (PROFSAT). All values remained below the recommended threshold of 0.85, thereby supporting discriminant validity.

#### 4.2.2 Model A

##### 4.2.2.1 Model specification and evaluation

**Model specification and identification:** The measurement models were specified according to the theoretically proposed instrument structure. The PCPI-S consists of 59 items loading on 17 constructs and 3 higher-order constructs. To reduce the number of parameters to be estimated, items were parcelled according to the specification of the 17 subfactors, resulting in a measurement model with 3 factors (Prerequisites “PRE”, Practice Environment “ENV”, Person-Centred Processes “PRO”) and 17 observed variables. The PCQ includes a total of 14 items and three subfactors (Safety “SAFE”, Everydayness “EVRY”, Community “COMM”). These three subfactors load on a higher-order factor (PCQ). Each factor (latent construct) was identified by at least three indicators. The factor variance was fixed to 1 to establish scale.

**Model estimation, evaluation, and respecification:** The fit statistics indicated an overall insufficient fit of the initial model to the data. The chi-square test of exact fit was significant [*χ*^2^ (425) = 1,948, *p* < 0.001], which indicates a deviation of the model from the data, and which is expected for models of this size (76). To improve model fit, the following adjustments were made: Two items were removed due to low factor loadings (< 0.5) (pcq_05 “A place where the staff use a language that the patients can understand”; pcpis_d11 m “The physical environment”); modification indices (≥ 10) suggested possible model improvements in the form of correlated errors. Eleven of these were theoretically justified and were implemented incrementally (supplementary material).

The adjusted model demonstrated good fit (*χ*^2^/df = 2.829, CFI = 0.930, TLI = 0.921, RMSEA = 0.060, SRMR = 0.068). Standardised factor loadings were sufficiently high and ranged from 0.499 (pcq_10) to 0.933 (pcpis_d9 m) (supplementary material). The factors were significantly correlated, with values between 0.380 (PRO, PCQ) and 0.845 (PRE, PRO). Fit statistics are provided in Table 4.

**Table 4:** Fit statistics for model A.

Model A	χ^2^ (*p*-value)	*df*	χ^2^/*df*	CFI	TLI	RMSEA	90% RMSEA	SRMR
Initial model	1,948 (*p* < 0.001)	425	4.584	0.845	0.830	0.085	0.081–0.089	0.083
Adjusted model	1,010 (*p* < 0.001)	357	2.829	0.930	0.921	0.060	0.056–0.065	0.068
Structural model	1,010 (*p* < 0.001)	357	2.829	0.930	0.921	0.060	0.056–0.065	0.068

*N* = 573. Structural model: Free parameters = 107; Estimation method: MLR; Missing values: FIML; Converged after 155 iterations.

**Figure 2:**
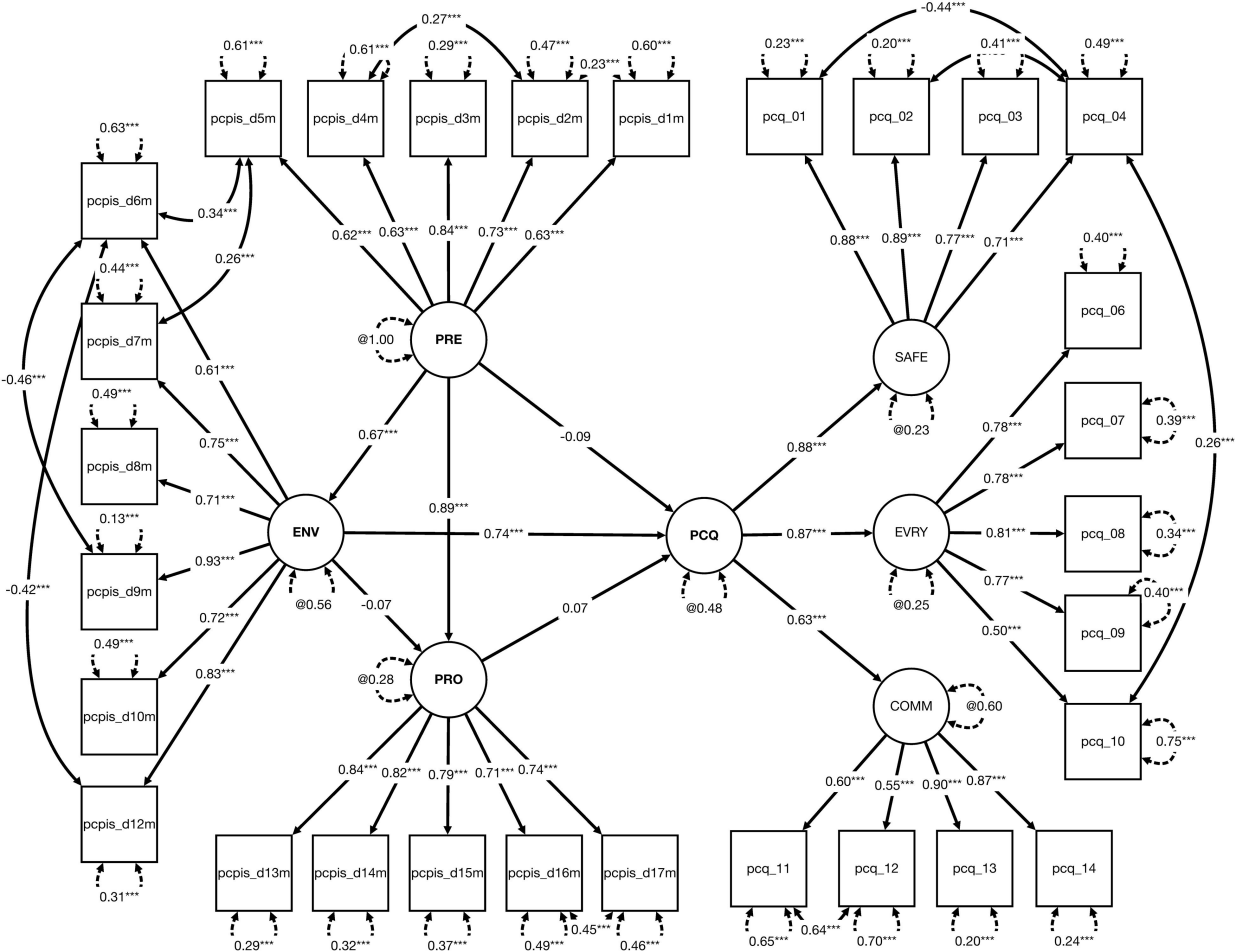
Path diagram for structural equation model A, displaying standardised parameter estimates.

**Structural model**: The structural model A (Figure 2) aims to predict the Person-Centred Climate (PCQ) based on nursing prerequisites (PRE), the practice environment (ENV), and person-centred processes (PRO). All three factors were modelled as direct influencing factors on the Person-Centred Climate. Additionally, indirect relationships were specified: between prerequisites and person-centred climate, mediated by the practice environment; between prerequisites and person-centred climate, mediated by person-centred processes; between prerequisites and person-centred climate, mediated by both environment and person-centred processes. A further indirect relationship, derived from the underlying theoretical framework (80), was modelled between prerequisites and person-centred processes, mediated by the practice environment.

The fit statistics indicate an overall satisfactory fit of the structural model to the data (Table 4). Tables of variances and covariances, as well as residual covariances and correlations, are provided in the supplementary material.

##### 4.2.2.2 Model interpretation

The model explains 51.8% of the variance in person-centred climate (PCQ). For person-centred processes (PRO), the explained variance is 71.7%, and 44.3% for the practice environment (ENV).

**Direct effects**: The results show a strong, significant positive association between the practice environment (ENV) and the person-centred climate (PCQ) (*β* = 0.74, *p* < 0.001). Other significant direct relationships were observed between prerequisites (PRE) and person-centred processes (PRO) (*β* = 0.89, *p* < 0.001) and between prerequisites and the practice environment (*β* = 0.67, *p* < 0.001).

No significant associations were found between prerequisites and person-centred climate, person-centred processes and person-centred climate, and person-centred processes and practice environment. Parameter estimates for direct effects in model A are provided in Table 5.

**Table 5:** Parameter estimates for direct effects in model A.

				95% Confidence intervals				
Dep	Pred	Estimate	SE	Lower	Upper	*β*	z	*p*
PCQ	PRE	−0.1304	0.1796	−0.4824	0.2216	−0.0905	−0.726	0.468
PCQ	ENV	0.7995	0.0973	0.6087	0.9903	0.7435	8.213	< .001
PCQ	PRO	0.0517	0.0758	−0.0968	0.2003	0.0675	0.683	0.495
PRO	PRE	1.6761	0.2821	1.1233	2.229	0.8916	5.942	< .001
PRO	ENV	−0.0978	0.0883	−0.2709	0.0753	−0.0697	−1.107	0.268
ENV	PRE	0.8917	0.08	0.7349	1.0484	0.6655	11.149	< .001

Dep, dependent variable; Pred, predictor variable; SE, standard error; *β*, standardised path coefficient.

**Indirect effects**: A significant indirect association (*β* = 0.50, *p* < 0.001) between prerequisites (PRE) and person-centred climate (PCQ) through the practice environment (ENV) was observed. This indicates that stronger prerequisites contribute to a more positive person-centred climate via a supportive practice environment, highlighting the central role of the practice environment as a mediating factor.

No other significant relationships were found in this model specification.

Parameter estimates for all indirect effects are provided in the supplementary material.

#### 4.2.3 Model B

##### 4.2.3.1 Model specification and evaluation

**Model specification and identification**: The measurement model was specified according to the theoretically proposed instrument structure. The Person-Centred Climate Questionnaire (PCQ) contains 14 items across three factors, Safety (“SAFE”, 5 items), Everydayness (“EVRY”, 5 items), and Community (“COMM”, 4 items). These three subfactors load onto a higher-order factor (PCQ). The Moral Distress Scale (MDS) contains 12 items unidimensional. Cool Down Index (CDI) comprises 9 items across two factors, Emotional Distancing (“EMODIST”, 6 items) and Emotional Withdrawal (“EMOWITH”, 3 items), which together form a higher-order factor (CO). The Job Satisfaction (JS) subscale of the NCI contains 10 items across two factors, Personal Satisfaction (“PERSSAT”, 5 items) and Professional Satisfaction (“PROFSAT”, 5 items). Intention to Leave (IL) contains 3 items. Each factor (latent construct) was identified by at least three indicators, and the factor variance was fixed to 1 to establish scale.

**Model estimation, evaluation, and respecification**: Although three of the relevant fit indices fall within the acceptable range (*χ*^2^/df = 2.757, RMSEA = 0.069, SRMR = 0.061), the fit statistics indicate an overall insufficient fit of this initial model to the data (Table 6). To improve model fit, the following adjustments were made: Three items were removed due to low factor loadings (< 0.5) (mds_dist_04 “Avoid taking action when I learn that a physician or nurse colleague has made a medical error and not reported it.”, mds_dist_07 “Ignore situations of suspected patient abuse by caregivers.”, nci_16 “The amount of time I spend on administration”); modification indices (≥ 10) suggested possible improvements in the form of correlated errors. Eight of these were theoretically justified and incrementally implemented (supplementary material). A cross-loading of one Cool Down item on Intention to Leave was allowed (cdi_07: “In dealing with the persons I am responsible to care for, I notice that I increasingly think how nice it would be to pack it all in”).

**Table 6:** Fit statistics for model B.

Model B	χ^2^ (*p*-value)	*df*	χ^2^/*df*	CFI	TLI	RMSEA	90% RMSEA	SRMR
Initial model	2,931 (*p* < 0.001)	1,063	2.757	0.865	0.857	0.059	0.056–0.061	0.061
Adjusted model	1,852 (*p* < 0.001)	876	2.114	0.926	0.920	0.047	0.044–0.050	0.054
Structural model	1,856 (*p* < 0.001)	878	2.114	0.926	0.920	0.047	0.044–0.050	0.055

*N* = 573. Structural model: free parameters = 156; Estimation method: MLR; Missing values: FIML; Converged after 218 iterations.

The adjusted model demonstrated good fit (*χ*^2^/df = 2.114, CFI = 0.926, TLI = 0.920, RMSEA = 0.047, SRMR = 0.054). Standardised factor loadings range from 0.519 (pcq_10) to 0.948 (nci_07). Item cdi_07 was theoretically justified to load on two factors to optimise model fit and shows a significant loading of 0.377 on IL. The latent variable EMODIST, a subscale of the Cool Down Index, does not show significant factor loadings in the two-factor specification. This is likely due to substantial shared variance with item cdi_07 via the cross-loading, which reduces the variance that can be uniquely attributed to EMODIST. Nevertheless, the model remains theoretically meaningful. A one-factor solution was explored as an alternative, showing significant factor loadings for all items.

The factors are significantly correlated, with values ranging from 0.36 (MD, IL) to 0.85 (JS, PCQ). Fit statistics are reported in Table 6.

**Figure 3:**
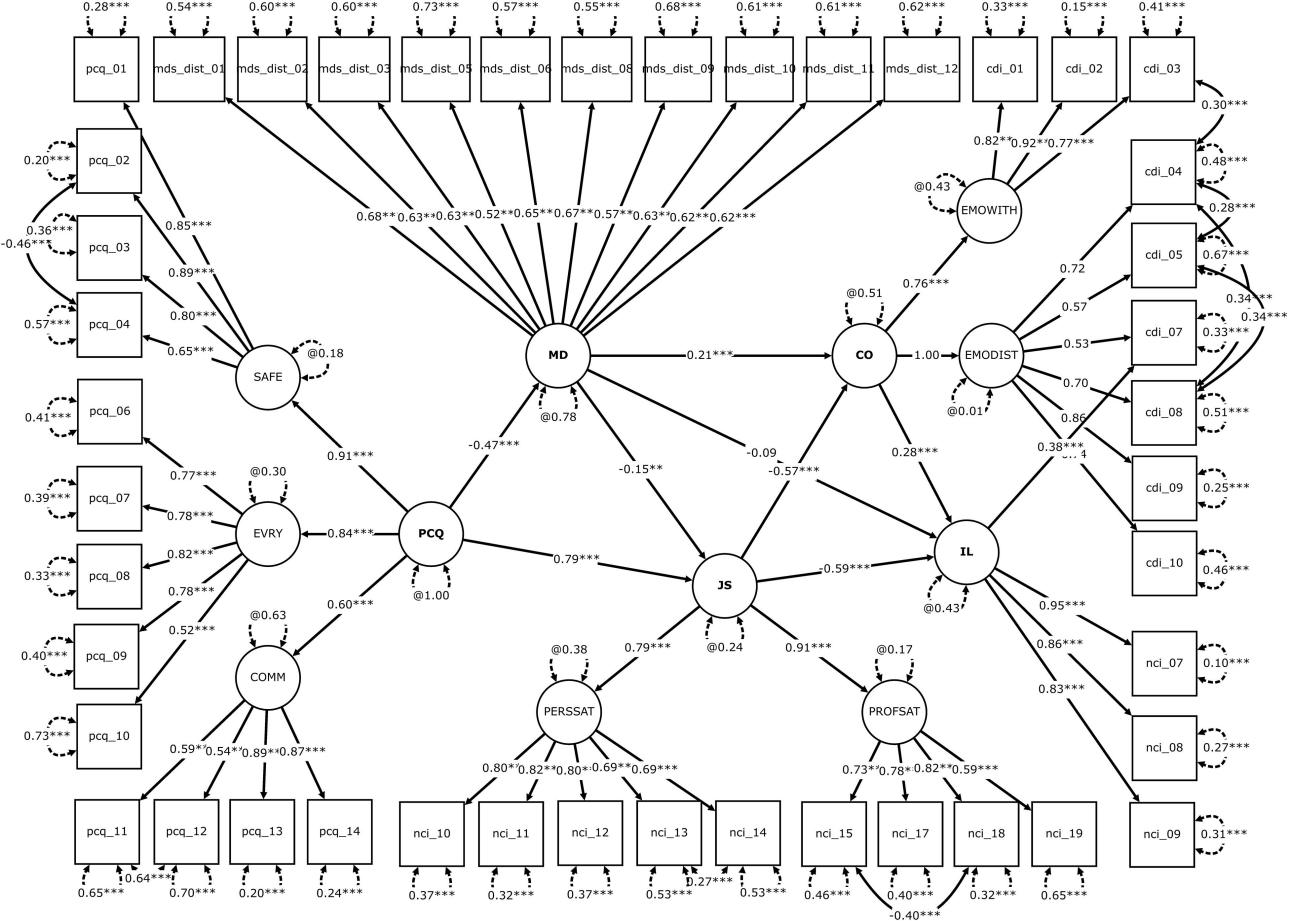
Path diagram for structural equation model B, displaying standardised parameter estimates.

**Structural model**: The structural Model B (Figure 3) was specified according to the hypothesised relationships. The model aims to predict intention to leave (IL) based on psychosocial climate (PCQ), moral distress (MD), Coolout (CO), and job satisfaction (JS). MD, CO, and JS were modelled as direct predictors of IL. Additionally, indirect relationships were specified between PCQ and IL, mediated by MD and CO; between PCQ and IL, mediated by JS; between JS and IL, mediated by CO; and between PCQ and IL, mediated by MD. The model also specifies multi-step paths between PCQ and IL.

The fit statistics indicate an overall satisfactory fit of the structural model to the data (Table 6). Tables of variances and covariances, as well as residual covariances and correlations, are provided in the supplementary material.

##### 4.2.3.2 Model interpretation

The model explains 57.3% of the variance in intention to leave (IL); 76.4% of job satisfaction (JS); 48.9% of coolout (CO); and 21.8% of moral distress (MD).

**Direct effects**: A positive association was found between coolout (CO) and intention to leave (IL) (*β* = 0.28, *p* < 0.001), as well as a negative association between job satisfaction (JS) and intention to leave (*β* = −0.59, *p* < 0.001). The association between moral distress (MD) and intention to leave was negative and nonsignificant (*β* = −0.09, *p* = 0.069). Higher MD values are associated with higher CO values (*β* = 0.21, *p* < 0.001), while higher job satisfaction is associated with lower CO values (*β* = −0.57, *p* < 0.001). A stronger person-centred climate (PCQ) is associated with lower moral distress (*β* = −0.47, *p* < 0.001) and higher job satisfaction (*β* = 0.79, *p* < 0.001). Lower moral distress is also associated with higher job satisfaction (*β* = −0.15, *p* = 0.003). Parameter estimates for direct effects in model B are provided in Table 7.

**Table 7:** Parameter estimates for direct effects in model B.

				95% Confidence intervals				
Dep	Pred	Estimate	SE	Lower	Upper	*β*	z	*p*
IL	CO	0.303	0.0701	0.165	0.43991	0.2765	4.32	< .001
IL	MD	−0.123	0.0678	−0.256	0.00952	−0.0911	−1.82	0.069
IL	JS	−0.44	0.0621	−0.561	−0.31808	−0.5911	−7.08	< .001
CO	MD	0.26	0.0664	0.13	0.39013	0.2102	3.92	< .001
CO	JS	−0.385	0.0505	−0.484	−0.28582	−0.566	−7.62	< .001
MD	PCQ	−0.528	0.0613	−0.648	−0.40777	−0.4669	−8.61	< .001
JS	PCQ	1.63	0.1941	1.249	2.01041	0.7921	8.4	< .001
JS	MD	−0.278	0.0948	−0.464	−0.09254	−0.1529	−2.94	0.003

Dep, dependent variable; Pred, predictor variable; SE, standard error; *β*, standardised path coefficient.

**Indirect effects**: Several significant indirect relationships were observed. The largest association was the indirect negative association between person-centred climate (PCQ) and intention to leave (IL) via job satisfaction (JS) (*β* = −0.468, *p* < 0.001). This suggests that a stronger person-centred climate contributes to lower intention to leave by increasing job satisfaction. The second largest association concerns job satisfaction (JS), which is negatively associated with intention to leave (IL) via Coolout (CO) (*β* = −0.156, *p* < 0.001). This association indicates that higher job satisfaction is associated with lower intention to leave by affecting the coolout process. Another indirect negative association (*β* = −0.124, *p* < 0.001) between person-centred climate (PCQ) and intention to leave (IL) suggests that a stronger person-centred climate reduces intention to leave by both increasing job satisfaction and influencing the coolout process. Parameter estimates for the remaining indirect effects are provided in the supplementary material.

## 5 Discussion

### 5.1 Summary of findings

The aim of this study was to test a theoretical model examining the impact of the practice environment on person-centred climate (Model A), and person-centred climate on turnover intention through job satisfaction, moral distress and coolout (Model B). Following a review of the literature and theory synthesis, a cross-sectional survey (*N* = 573) was conducted in the autumn of 2024.

Validated instruments were used to assess the relevant theoretical constructs, including person-centred practice (PCPI-S), person-centred climate (PCQ-S), moral distress (MDS), coolout (CDI), as well as job satisfaction and intention to leave (subscales of the NCI). Sociodemographic data were also collected. The theoretical model was tested using structural equation modelling. Because of the complexity of the model, two separate models were specified: Model A posited that nurses’ prerequisites, the practice environment, and person-centred processes predict the perceived person-centred climate; Model B examined the extent to which the person-centred climate influences intention to leave, mediated by moral distress, emotional distancing (coolout), and job satisfaction.

Both models showed acceptable fit following theory-informed adjustments. In both cases, a significant improvement of model fit was achieved through theoretically justified modifications, including correlated error terms and the removal of individual items with low factor loadings.

In Model A, two items related to the comprehensibility of language (part of the PCQ-S construct “Climate of Safety”) and aspects of the physical environment (part of the PCPI-S construct “The Practice Environment”) were excluded due to low factor loadings. In Model B, two items from the Moral Distress Scale (unreported medical errors and suspected patient abuse), as well as an item reflecting administrative workload as part of professional satisfaction, were removed for the same reason. These modifications improved model fit and were undertaken with careful consideration of theoretical coherence and potential implications for construct representation. All affected scales retained a sufficient number of items to ensure appropriate representation of the underlying theoretical constructs.

In Model A, predictors explained 51.8% of the variance in person-centred climate. The practice environment emerged as the sole significant predictor (*β* = 0.74, *p* < 0.001) and mediator (*β* = 0.50, *p* < 0.001) between nurses’ prerequisites and a person-centred climate. In Model B, predictors explained 57.3% of the variance in intention to leave. A person-centred climate was indirectly associated with a lower intention to leave via job satisfaction (*β* = −0.468, *p* < 0.001) and, to a lesser extent, through the combination of job satisfaction and coolout (*β* = −0.124, *p* < 0.001). The indirect association via moral distress was negligible and non-significant (*β* = 0.043, *p* = 0.067).

### 5.2 Integration with existing evidence and theory

The findings provide partial support for the proposed hypotheses, suggesting that a person-centred climate is associated with nurse retention and emphasising the role of supportive environments for nurse well-being, including an association with low moral distress and high job satisfaction. The strong associations between prerequisites, practice environment, person-centred climate, nurse satisfaction and turnover intention are consistent with previous findings (14, 80–82).

Coolout, i.e., emotional exhaustion and withdrawal, was identified as a significant mediating factor between a person-centred climate, job satisfaction, and turnover intention, which adds important nuance to the discussion. A large body of turnover intention research has focused on burnout (83). Research on coolout, at least under this label, by contrast, is relatively new, with significant theoretical and empirical contributions made by Büssing and colleagues (39, 40, 61) as well as Kersting (41, 53). At the same time, the interpretation of coolout as a distinct mediating construct should be approached with caution. In the present model, the inclusion of a cross-loading between a CDI item and intention to leave, while meaningful at the item level, as well as unstable factor loading behaviour in one CDI subscale, points to potential limitations in construct stability and possible conceptual overlap with turnover intention. This suggests that the empirical distinction between coolout and related constructs may not yet be fully established. Emotional exhaustion can manifest as an impersonal and cynical attitude towards patients (39). This is an alarming state of being, as it stands in a stark contrast to professional values of nursing. However, given the measurement-related limitations observed in this study, the role of coolout as a mediator should be considered preliminary and warrants further validation in future research.

Other hypotheses were not supported by the data, concerning determinants of a person-centred climate and their interrelations on the one hand, and the association between moral distress and intention to leave as well as its mediating role, on the other.

First, contrary to expectations, the association between moral distress and intention to leave was negligible and non-significant. While a person-centred climate was associated with low moral distress, and low moral distress was associated with high job satisfaction, it was not significantly associated with intention to leave in this study. Moral distress occurs when nurses are prevented from acting in accordance with their moral and ethical principles (32). Moral distress is assumed to be an inherent aspect of healthcare that cannot be entirely avoided (31). Participants in this study reported moderate to low levels of both moral distress and intention to leave. Higher moral distress is consistently associated with stronger intention to leave, among many other negative consequences (34, 84, 85). However, recent research suggests that the role of moral distress may be overstated, as it can be understood as an expression of moral sensitivity, which is considered indispensable in this field (30), drawing attention to values and norms and reminding nurses of what they stand for (31). Indeed, recent findings suggest that nurses with well-developed moral sensitivity are more inclined to deliver person-centred care (86), but they may also experience increased moral distress (87). The present findings align with these more recent insights. Accordingly, the goal is not the complete avoidance of moral distress but to support the development of effective coping strategies that allow moral sensitivity to be preserved while healthy boundaries are maintained (87). Coolout, as a form of emotional withdrawal, can be considered a coping strategy, albeit a maladaptive one. Deliberate efforts are recommended to counteract such patterns by supporting the development of constructive coping strategies.

Second, there was no significant association between person-centred processes and a person-centred climate. Person-centred processes refer to aspects of the interaction between nurses and patients or their families (46), while person-centred climate refers to how nurses experience the psychosocial aspects of their work environment (59). Since the latter largely depends on attitudes and actions of leadership and colleagues, rather than interaction with patients, this might explain the lack of association.

Finally, while the practice environment had a significant association with person-centred climate and mediated its relationship with prerequisites, it had neither a direct association to person-centred processes, nor did it mediate the relationship between prerequisites of nurses and person-centred processes, as proposed in the Person-Centred Practice Framework (46, 80). While this aligns with findings from the original authors (80), another study did report a significant association between these constructs (82). The reasons for these differences in association remain to be further explored.

### 5.3 Implications for research and practice

The findings may serve as a tentative basis for informing retention strategies at both the meso and micro levels, including the development of monitoring tools and supplementary measures to support practice development for teams and individual practitioners, although further validation is required.

The findings may inform the development of brief monitoring instruments to assess factors associated with turnover intention and retention-related outcomes at the ward or team level. The tested model suggests that person-centred climate and related aspects of the practice environment are associated with patterns involving moral distress, emotional withdrawal, job satisfaction, and turnover intention. As person-centred climate may represent a less stigmatising aspect of the work environment to assess, future pulse-monitoring approaches could combine selected change-sensitive items assessing person-centred climate with single-item indicators of moral distress and coolout-related emotional withdrawal. Such applications should be considered preliminary implications of the present findings rather than validated practice recommendations. Given the cross-sectional design of this study, longitudinal research is needed to determine whether changes in these indicators are associated with subsequent changes in turnover-related outcomes and whether they can meaningfully support organisational monitoring efforts.

### 5.4 Strengths and limitations

This study provides empirical evidence for the association between person-centred practice environments, intention to leave and associated factors. Nevertheless, several limitations should be considered when interpreting the findings.

This study employed a simple structural equation model using cross-sectional data. Although the specified paths are theoretically intended to reflect a process, empirically they can only be interpreted as associations. Accordingly, causal inferences should be drawn with caution.

Due to the overall model complexity, the analysis was conducted using a split-model approach. While this has facilitated model estimation, it may have limited the ability to capture the full complexity of the underlying relationships within a single comprehensive model.

Although the achieved sample size exceeded the predefined target and can be considered adequate, model complexity may have limited the precision of parameter estimates.

Although the study was conducted within one healthcare organisation, this organisation represents the largest healthcare provider in Austria and comprises multiple care settings. Participants were recruited from ten different institutions, including hospitals, long-term care and psychiatric care facilities. Thus, while the sampling strategy limits statistical generalisability, the sample reflects a broad organisational context rather than a single-site setting.

Potential clustering effects in the sample at the facility level could not be statistically accounted for because cluster-robust estimators were not available. As a result, standard errors may have been underestimated, given that nurses within same facility are likely to be more similar to one another, thereby violating the assumption of independence of observations. While the reported parameter estimates remain unaffected, the inferential statistics (standard errors, confidence intervals, p-values) should therefore be interpreted with due caution.

Although item parcelling was applied to the PCPI-S based on established theoretical and empirical considerations to enhance model parsimony, it may have limited the detection of item-level misfit and should therefore be interpreted with caution.

Discriminant validity was limited for some latent construct pairs. The high latent association between person-centred climate and job satisfaction suggests that these constructs were closely related in the present sample. This is theoretically plausible, as both constructs reflect evaluative perceptions of the work environment. However, the strength of this association raises concerns regarding discriminant validity and limits the interpretation of unique effects involving these constructs.

A further limitation concerns the choice of estimation method. Although estimators such as WLSMV and DWLS are often recommended for ordinal indicators, these methods did not yield stable and admissible solutions in the present complex higher-order SEM. Therefore, MLR was used as a robust estimator, providing robust standard errors and fit statistics under non-normality while accommodating mixed indicator types in complex higher-order models. Model A used questionnaire items with 5- or 6-point Likert-type response scales. In the SEM, indicators included both individual items and parcel/subscale scores for larger established instruments. Parcels were created according to predefined theoretical subscale structures and treated as approximately continuous. Model B consisted of 5- or 6-point ordinal Likert-type and frequency indicators. Although MLR is commonly applied in such contexts, alternative estimators may have yielded different parameter estimates. The results should therefore be interpreted in light of this pragmatic decision regarding model estimation.

Model respecification (item deletion, correlated residuals, cross-loading) was undertaken based on both statistical indicators (modification indices) and theoretical considerations. While these adjustments improved model fit, they may have implications for construct representation, which should be considered in the interpretation of our findings.

Another limitation of this study concerns the involvement of the commissioning organisation and the declared conflict of interest, which may influence perceptions of research independence. Given that the study was commissioned and funded by the organisation providing the study sites, and that two authors have institutional links to this organisation, it is important to consider how these relationships may have shaped certain aspects of the research process. While access to study sites was necessarily facilitated by the commissioning organisation, site selection itself was based on predefined representation criteria and determined independently by the research team. Close collaboration with practice partners is a core feature of person-centred research and was important for ensuring the relevance and applicability of the findings. Beyond this, the commissioning context did not influence the choice of instruments, the analysis, or the interpretation of the data. A key measure to safeguard scientific independence was a formal compliance agreement established prior to the start of the study, ensuring independence in study design, data analysis, interpretation of results, and the decision to publish.

Finally, while turnover intention is a recognised predictor of actual turnover, it remains a proxy measure, and therefore the inclusion of actual turnover data is necessary to support more robust conclusions.

## 6 Conclusion

The findings provide evidence for the association between a person-centred climate and turnover intention, highlighting the relationship between supportive environments, nurse well-being and hence effective nursing practice, and adding nuance with the theoretical concept of coolout (emotional withdrawal and distancing). A person-centred climate is associated with high job satisfaction, low emotional withdrawal, and low turnover intention. A person-centred climate is associated with various factors within the practice environment, including effective staff relationships, shared decision-making systems, and an appropriate skill mix. The deliberate development of supportive person-centred environments is therefore a key element in practice development and staff retention to address challenges related to workforce developments. Future research should further explore mediating mechanisms and develop tools and interventions to inform policy action as well as practice development and retention efforts. While the model provides preliminary support for associations among the examined constructs, further validation using longitudinal, multisite research designs, and actual turnover data is required.

## Data Availability

The dataset presented in this article is not readily available because the commissioning organisation maintains a legitimate interest in keeping the data confidential. Requests to access the dataset should be directed to hanna.mayer@kl.ac.at.
