# Supplementary material for: Person-centred environments and nurses’ turnover intention: examining their relationship using structural equation modelling
Source: Front Health Serv. 2026 Jul 15;6:1816479. doi: 10.3389/frhs.2026.1816479 (PMC13415943; doi:10.3389/frhs.2026.1816479)
Supplement: Supplementary file 1 [file Table1.docx]

Supplementary Material

# Supplementary Tables

## Latent variable correlations

## Model A

|  | SAFE | EVRY | COMM | PRE | ENV | PRO | PCQ |
| --- | --- | --- | --- | --- | --- | --- | --- |
| SAFE | 1.000 |  |  |  |  |  |  |
| EVRY | 0.775 | 1.000 |  |  |  |  |  |
| COMM | 0.570 | 0.550 | 1.000 |  |  |  |  |
| PRE | 0.387 | 0.374 | 0.275 | 1.000 |  |  |  |
| ENV | 0.666 | 0.643 | 0.472 | 0.662 | 1.000 |  |  |
| PRO | 0.338 | 0.326 | 0.240 | 0.815 | 0.531 | 1.000 |  |
| PCQ | 0.896 | 0.865 | 0.636 | 0.432 | 0.743 | 0.377 | 1.000 |

*Notes*. Model-implied latent correlations based on the initial measurement model specification. SAFE “climate of safety”, EVRY “climate of everydayness”, COMM “climate of community”, PRE “nurse prerequisites”, ENV “practice environment”, PRO “person-centred processes”, PCQ “person-centred climate”. SAFE, EVRY, and COMM are first-order latent constructs loading on the higher-order factor PCQ.

## Model B

|  | SAFE | EVRY | COMM | EMODIST | EMOWITH | PERSSAT | PROFSAT | MD | IL | CO | JS | PCQ |
| --- | --- | --- | --- | --- | --- | --- | --- | --- | --- | --- | --- | --- |
| SAFE | 1.000 |  |  |  |  |  |  |  |  |  |  |  |
| EVRY | 0.776 | 1.000 |  |  |  |  |  |  |  |  |  |  |
| COMM | 0.575 | 0.532 | 1.000 |  |  |  |  |  |  |  |  |  |
| EMODIST | -0.524 | -0.485 | -0.359 | 1.000 |  |  |  |  |  |  |  |  |
| EMOWITH | -0.392 | -0.363 | -0.269 | 0.738 | 1.000 |  |  |  |  |  |  |  |
| PERSSAT | 0.611 | 0.565 | 0.419 | -0.543 | -0.407 | 1.000 |  |  |  |  |  |  |
| PROFSAT | 0.719 | 0.665 | 0.493 | -0.639 | -0.478 | 0.757 | 1.000 |  |  |  |  |  |
| MD | -0.426 | -0.395 | -0.292 | 0.502 | 0.376 | -0.416 | -0.490 | 1.000 |  |  |  |  |
| IL | -0.598 | -0.553 | -0.410 | 0.648 | 0.485 | -0.566 | -0.666 | 0.365 | 1.000 |  |  |  |
| CO | -0.528 | -0.488 | -0.362 | 0.993 | 0.743 | -0.547 | -0.644 | 0.505 | 0.653 | 1.000 |  |  |
| JS | 0.762 | 0.705 | 0.522 | -0.677 | -0.507 | 0.802 | 0.944 | -0.519 | -0.706 | -0.682 | 1.000 |  |
| PCQ | 0.916 | 0.847 | 0.628 | -0.572 | -0.428 | 0.667 | 0.785 | -0.466 | -0.653 | -0.576 | 0.832 | 1.000 |

*Notes*. Model-implied latent correlations based on the initial measurement model specification. SAFE “climate of safety”, EVRY “climate of everydayness”, COMM “climate of community”, PRE “nurse prerequisites”, ENV “practice environment”, PRO “person-centred processes”, EMODIST “emotional distancing”, EMOWITH “emotional withdrawal”, PERSSAT “personal satisfaction”, PROFSAT “professional satisfaction”, MD “moral distress”, IL “intention to leave”, CO “coolout”, JS “job satisfaction”, PCQ “person-centred climate”. SAFE, EVRY, and COMM are first-order latent constructs loading on the higher-order factor PCQ. PERSSAT and PROFSAT load on the higher-order factor JS. EMODIST and EMOWITH load on the higher-order factor CO.

## Model respecification

Model respecification was informed by modification indices (>10); correlated error terms were specified incrementally between the following item pairs (A/B), guided by theoretical considerations. Items are presented in German, with English translations provided in square brackets.

Model A respecification

| Item A | Item B |
| --- | --- |
| pcq_11: “… ein Ort, an dem es für die Patient*innen leicht ist, mit ihren Lieben in Kontakt zu bleiben.” [*A place where it is easy for the patients to keep in contact with their loved ones.*] | pcq_12: “… ein Ort, an dem es für die Patient*innen einfach ist, Besuch zu empfangen.” [*A place where it is easy for the patients to receive visitors.*] |
| pcpis_d16m: “Professionell sorgend präsent sein” [*Being sympathetically present*] | pcpis_d17m: “Ganzheitliches Arbeiten” [*Working holistically*] |
| pcpis_d5m: “Klarheit über Werte und Überzeugungen haben” [*Clarity of beliefs and values*] | pcpis_d6m: “angemessener Skill-Mix” [*Appropriate skill mix*] |
| pcq_02: “… ein Ort, an dem ich mich als Person anerkannt fühle.” [*A place where I feel acknowledged as a person.*] | pcq_04: “… ein Ort, an dem die Patient*innen in sicheren Händen sind.” [*A place where the patients are in safe hands.*] |
| pcpis_d6m: “angemessener Skill-Mix” [*Appropriate skill mix*] | pcpis_d12m: “unterstützende Organisation” [*Supportive organisational systems*] |
| pcpis_d6m: “angemessener Skill-Mix” [*Appropriate skill mix*] | pcpis_d9m: “Machtverteilung (flache Hierachien)” [*Power sharing*] |
| pcpis_d5m: “Klarheit über Werte und Überzeugungen haben” [*Clarity of beliefs and values*] | pcpis_d7m: “gemeinsame Entscheidungsprozesse” [*Shared decision making systems*] |
| pcpis_d2m: “Entwickelte zwischenmenschliche Fähigkeiten haben” [*Developed interpersonal skills*] | pcpis_d4m: “sich selbst kennen” [*Knowing self*] |
| pcpis_d1m: “fachlich kompetent sein” [*Professionally competent*] | pcpis_d2m: “Entwickelte zwischenmenschliche Fähigkeiten haben” [*Developed interpersonal skills*] |
| pcq_04: “… ein Ort, an dem die Patient*innen in sicheren Händen sind.” [*A place where the patients are in safe hands.*] | pcq_10: “… ein Ort, der ordentlich und sauber ist.” [*A place which is neat and clean.*] |
| pcq_01: “… ein Ort, an dem ich mich willkommen fühle.” [*A place where I feel welcome.*] | pcq_04: “… ein Ort, an dem die Patient*innen in sicheren Händen sind.” [*A place where the patients are in safe hands.*] |

Model B respecification

| Variable A | Variable B |
| --- | --- |
| pcq_11: “… ein Ort, an dem es für die Patient*innen leicht ist, mit ihren Lieben in Kontakt zu bleiben.” [*A place where it is easy for the patients to keep in contact with their loved ones.*] | pcq_12: “… ein Ort, an dem es für die Patient*innen einfach ist, Besuch zu empfangen.” [*A place where it is easy for the patients to receive visitors.*] |
| pcq_02: “… ein Ort, an dem ich mich als Person anerkannt fühle.” [*A place where I feel acknowledged as a person.*] | pcq_04: “… ein Ort, an dem die Patient*innen in sicheren Händen sind.” [*A place where the patients are in safe hands.*] |
| cdi_04: “… dass ich oft gar nicht mehr die Geduld habe, ihnen zuzuhören.” [… *I often no longer have the patience to listen to them*.] | cdi_03: “… dass mir ihre persönlichen Probleme und Sorgen oft einfach zu viel werden.” [… *their personal problems and worries often simply become too much for me*.] |
| nci_13: “… dem Ausmaß an Herausforderungen in Ihrem Beruf.” [*The amount of challenge in my job.*] | nci_14: “… dem Ausmaß dessen, inwiefern Ihre Arbeit abwechslungsreich und interessant ist.” [*The extent to which my job is varied and interesting.*] |
| nci_15: “… der Art und Weise, wie es Ihnen möglich ist, zu pflegen und zu betreuen.” [*The way that I am able to care for patients.*] | nci_18: “… dem Ausmaß an Respekt und Fairness, welche die Führungskräfte Ihnen gegenüber zeigen.” [*The degree of respect and fair treatment I receive from my boss.*] |
| cdi_04: “… dass ich oft gar nicht mehr die Geduld habe, ihnen zuzuhören.” [… *I often no longer have the patience to listen to them*.] | cdi_08: “… dass mich einige von ihnen einfach nur nerven.” [… *some of them simply annoy me*.] |
| cdi_05: “… dass mir eigentlich egal ist, was diese von mir denken.“ [… *I largely don´t care what they think of me*.] | cdi_08: “… dass mich einige von ihnen einfach nur nerven.” [… *some of them simply annoy me*.] |
| cdi_04: “… dass ich oft gar nicht mehr die Geduld habe, ihnen zuzuhören.” [… *I often no longer have the patience to listen to them*.] | cdi_05: “… dass mir eigentlich egal ist, was diese von mir denken.” [… *I largely don´t care what they think of me*.] |

References

Büssing A, Falkenberg Z, Schoppe C, Recchia DR, Poier D. Work stress associated cool down reactions among nurses and hospital physicians and their relation to burnout symptoms. BMC Health Services Research. 2017;17(1):551.

Edvardsson D, Koch S, Nay R. Psychometric evaluation of the English language person‐centred climate questionnaire–staff version. Journal of nursing management. 2010;18(1):54-60.

McCance T, McCormack B. The Person-Centred Practice Framework. In: McCormack B, McCance T, Bulley C, Brown D, McMillan A, Martin S, editors. Fundamentals of Person-centred Healthcare Practice: John Wiley & Sons; 2021. p. 23-33.

Slater P, McCance T, McCormack B. The development and testing of the Person-centred Practice Inventory - Staff (PCPI-S). Int J Qual Health Care. 2017;29(4):541-7.

Slater P, McCormack B, Bunting B. The Development and Pilot Testing of an Instrument to Measure Nurses’ Working Environment: The Nursing Context Index. Worldviews on Evidence-Based Nursing. 2009;6(3):173-82.

Weis MLD, Wallner M, Köck-Hódi S, Hildebrandt C, McCormack B, Mayer H. German translation, cultural adaptation and testing of the Person-centred Practice Inventory - Staff (PCPI-S). Nurs Open. 2020;7(5):1400-11.

Wilfing D, Möhler R, Köpke S, Dichter MN. Person-Centred Climate Questionnaire – German version (PCQ-G) Benutzerhandbuch für die deutschsprachige Version. Köln 2022.

## Measurement model: factor loadings

Adjusted Model A

|  | | | | 95% Confidence Intervals | |  | | |
| --- | --- | --- | --- | --- | --- | --- | --- | --- |
| Latent | Observed | Estimate | SE | Lower | Upper | β | z | p |
| SAFE | pcq_01 | 0.476 | 0.0467 | 0.384 | 0.567 | 0.877 | 10.18 | <.001 |
|  | pcq_02 | 0.545 | 0.0522 | 0.443 | 0.647 | 0.892 | 10.44 | <.001 |
|  | pcq_03 | 0.452 | 0.0419 | 0.370 | 0.534 | 0.770 | 10.79 | <.001 |
|  | pcq_04 | 0.370 | 0.0406 | 0.291 | 0.450 | 0.714 | 9.12 | <.001 |
| EVRY | pcq_06 | 0.486 | 0.0422 | 0.403 | 0.569 | 0.775 | 11.52 | <.001 |
|  | pcq_07 | 0.533 | 0.0506 | 0.433 | 0.632 | 0.784 | 10.52 | <.001 |
|  | pcq_08 | 0.514 | 0.0492 | 0.418 | 0.611 | 0.813 | 10.45 | <.001 |
|  | pcq_09 | 0.494 | 0.0467 | 0.402 | 0.586 | 0.774 | 10.58 | <.001 |
|  | pcq_10 | 0.298 | 0.0332 | 0.233 | 0.363 | 0.499 | 8.99 | <.001 |
| COMM | pcq_11 | 0.508 | 0.0358 | 0.438 | 0.578 | 0.595 | 14.18 | <.001 |
|  | pcq_12 | 0.482 | 0.0408 | 0.402 | 0.562 | 0.545 | 11.81 | <.001 |
|  | pcq_13 | 0.706 | 0.0399 | 0.628 | 0.784 | 0.895 | 17.68 | <.001 |
|  | pcq_14 | 0.752 | 0.0387 | 0.676 | 0.828 | 0.871 | 19.44 | <.001 |
| PRE | pcpis_d1m | 0.383 | 0.0288 | 0.327 | 0.440 | 0.629 | 13.30 | <.001 |
|  | pcpis_d2m | 0.399 | 0.0270 | 0.346 | 0.452 | 0.730 | 14.80 | <.001 |
|  | pcpis_d3m | 0.513 | 0.0246 | 0.465 | 0.561 | 0.840 | 20.88 | <.001 |
|  | pcpis_d4m | 0.437 | 0.0348 | 0.369 | 0.505 | 0.625 | 12.54 | <.001 |
|  | pcpis_d5m | 0.491 | 0.0352 | 0.422 | 0.560 | 0.624 | 13.96 | <.001 |
| ENV | pcpis_d6m | 0.288 | 0.0220 | 0.245 | 0.331 | 0.610 | 13.11 | <.001 |
|  | pcpis_d7m | 0.513 | 0.0259 | 0.463 | 0.564 | 0.746 | 19.86 | <.001 |
|  | pcpis_d8m | 0.499 | 0.0310 | 0.438 | 0.560 | 0.711 | 16.10 | <.001 |
|  | pcpis_d9m | 0.656 | 0.0307 | 0.596 | 0.716 | 0.933 | 21.39 | <.001 |
|  | pcpis_d10m | 0.376 | 0.0199 | 0.337 | 0.415 | 0.715 | 18.87 | <.001 |
|  | pcpis_d12m | 0.620 | 0.0322 | 0.557 | 0.683 | 0.833 | 19.23 | <.001 |
| PRO | pcpis_d13m | 0.282 | 0.0310 | 0.221 | 0.342 | 0.843 | 9.10 | <.001 |
|  | pcpis_d14m | 0.339 | 0.0380 | 0.265 | 0.413 | 0.825 | 8.93 | <.001 |
|  | pcpis_d15m | 0.243 | 0.0280 | 0.188 | 0.298 | 0.792 | 8.66 | <.001 |
|  | pcpis_d16m | 0.220 | 0.0231 | 0.174 | 0.265 | 0.712 | 9.52 | <.001 |
|  | pcpis_d17m | 0.257 | 0.0273 | 0.204 | 0.311 | 0.738 | 9.41 | <.001 |
| PCQ | SAFE | 1.259 | 0.1460 | 0.973 | 1.545 | 0.876 | 8.62 | <.001 |
|  | EVRY | 1.197 | 0.1549 | 0.893 | 1.500 | 0.865 | 7.73 | <.001 |
|  | COMM | 0.564 | 0.0693 | 0.428 | 0.700 | 0.631 | 8.13 | <.001 |

 Adjusted Model B

|  | | | | 95% Confidence Intervals | |  | | |
| --- | --- | --- | --- | --- | --- | --- | --- | --- |
| Latent | Observed | Estimate | SE | Lower | Upper | β | z | p |
| SAFE | pcq_01 | 0.4026 | 0.0457 | 0.313 | 0.492 | 0.846 | 8.812 | <.001 |
|  | pcq_02 | 0.4783 | 0.0541 | 0.372 | 0.584 | 0.894 | 8.842 | <.001 |
|  | pcq_03 | 0.4128 | 0.0451 | 0.324 | 0.501 | 0.802 | 9.150 | <.001 |
|  | pcq_04 | 0.2975 | 0.0326 | 0.234 | 0.361 | 0.652 | 9.132 | <.001 |
| EVRY | pcq_06 | 0.5222 | 0.0389 | 0.446 | 0.598 | 0.769 | 13.422 | <.001 |
|  | pcq_07 | 0.5751 | 0.0475 | 0.482 | 0.668 | 0.782 | 12.106 | <.001 |
|  | pcq_08 | 0.5601 | 0.0445 | 0.473 | 0.647 | 0.818 | 12.592 | <.001 |
|  | pcq_09 | 0.5360 | 0.0429 | 0.452 | 0.620 | 0.775 | 12.509 | <.001 |
|  | pcq_10 | 0.3383 | 0.0340 | 0.272 | 0.405 | 0.519 | 9.953 | <.001 |
| COMM | pcq_11 | 0.5205 | 0.0373 | 0.447 | 0.594 | 0.595 | 13.955 | <.001 |
|  | pcq_12 | 0.4945 | 0.0418 | 0.413 | 0.577 | 0.545 | 11.821 | <.001 |
|  | pcq_13 | 0.7222 | 0.0388 | 0.646 | 0.798 | 0.892 | 18.616 | <.001 |
|  | pcq_14 | 0.7749 | 0.0379 | 0.701 | 0.849 | 0.874 | 20.424 | <.001 |
| EMODIST | cdi_04 | 0.0877 | 0.2573 | -0.417 | 0.592 | 0.724 | 0.341 | 0.733 |
|  | cdi_05 | 0.0671 | 0.1972 | -0.319 | 0.454 | 0.572 | 0.340 | 0.734 |
|  | cdi_07 | 0.0747 | 0.2188 | -0.354 | 0.504 | 0.526 | 0.341 | 0.733 |
|  | cdi_08 | 0.0916 | 0.2692 | -0.436 | 0.619 | 0.701 | 0.340 | 0.734 |
|  | cdi_09 | 0.1241 | 0.3641 | -0.589 | 0.838 | 0.864 | 0.341 | 0.733 |
|  | cdi_10 | 0.1044 | 0.3062 | -0.496 | 0.704 | 0.738 | 0.341 | 0.733 |
| EMOWITH | cdi_01 | 0.8857 | 0.0624 | 0.763 | 1.008 | 0.820 | 14.192 | <.001 |
|  | cdi_02 | 0.9611 | 0.0621 | 0.839 | 1.083 | 0.920 | 15.467 | <.001 |
|  | cdi_03 | 0.6998 | 0.0492 | 0.603 | 0.796 | 0.766 | 14.220 | <.001 |
| PERSSAT | nci_10 | 0.4887 | 0.0386 | 0.413 | 0.564 | 0.796 | 12.662 | <.001 |
|  | nci_11 | 0.4846 | 0.0365 | 0.413 | 0.556 | 0.822 | 13.280 | <.001 |
|  | nci_12 | 0.4210 | 0.0326 | 0.357 | 0.485 | 0.796 | 12.899 | <.001 |
|  | nci_13 | 0.4406 | 0.0336 | 0.375 | 0.507 | 0.688 | 13.100 | <.001 |
|  | nci_14 | 0.4097 | 0.0326 | 0.346 | 0.474 | 0.688 | 12.561 | <.001 |
| PROFSAT | nci_15 | 0.3115 | 0.0383 | 0.236 | 0.387 | 0.734 | 8.134 | <.001 |
|  | nci_17 | 0.3458 | 0.0415 | 0.265 | 0.427 | 0.776 | 8.340 | <.001 |
|  | nci_18 | 0.4404 | 0.0545 | 0.334 | 0.547 | 0.825 | 8.087 | <.001 |
|  | nci_19 | 0.2559 | 0.0313 | 0.194 | 0.317 | 0.590 | 8.164 | <.001 |
| MD | mds_dist_01 | 0.8324 | 0.0399 | 0.754 | 0.911 | 0.676 | 20.844 | <.001 |
|  | mds_dist_02 | 0.7620 | 0.0477 | 0.669 | 0.855 | 0.634 | 15.980 | <.001 |
|  | mds_dist_03 | 0.7333 | 0.0455 | 0.644 | 0.822 | 0.631 | 16.120 | <.001 |
|  | mds_dist_05 | 0.5786 | 0.0554 | 0.470 | 0.687 | 0.520 | 10.447 | <.001 |
|  | mds_dist_06 | 0.8657 | 0.0473 | 0.773 | 0.958 | 0.654 | 18.295 | <.001 |
|  | mds_dist_08 | 0.8300 | 0.0485 | 0.735 | 0.925 | 0.670 | 17.115 | <.001 |
|  | mds_dist_09 | 0.7988 | 0.0569 | 0.687 | 0.910 | 0.567 | 14.040 | <.001 |
|  | mds_dist_10 | 0.7588 | 0.0555 | 0.650 | 0.868 | 0.627 | 13.672 | <.001 |
|  | mds_dist_11 | 0.8463 | 0.0522 | 0.744 | 0.949 | 0.622 | 16.203 | <.001 |
|  | mds_dist_12 | 0.7710 | 0.0491 | 0.675 | 0.867 | 0.617 | 15.694 | <.001 |
| IL | nci_07 | 0.8093 | 0.0460 | 0.719 | 0.899 | 0.947 | 17.579 | <.001 |
|  | nci_08 | 0.6981 | 0.0419 | 0.616 | 0.780 | 0.856 | 16.665 | <.001 |
|  | nci_09 | 0.7747 | 0.0424 | 0.692 | 0.858 | 0.831 | 18.262 | <.001 |
|  | cdi_07 | 0.3873 | 0.0495 | 0.290 | 0.484 | 0.376 | 7.823 | <.001 |
| CO | EMOWITH | 0.8256 | 0.0872 | 0.655 | 0.997 | 0.756 | 9.464 | <.001 |
|  | EMODIST | 7.9036 | 23.4960 | -38.148 | 53.955 | 0.996 | 0.336 | 0.737 |
| JS | PERSSAT | 0.6256 | 0.0879 | 0.453 | 0.798 | 0.790 | 7.115 | <.001 |
|  | PROFSAT | 1.0790 | 0.1808 | 0.725 | 1.433 | 0.912 | 5.968 | <.001 |
| PCQ | SAFE | 2.1424 | 0.2783 | 1.597 | 2.688 | 0.906 | 7.698 | <.001 |
|  | EVRY | 1.5451 | 0.1535 | 1.244 | 1.846 | 0.840 | 10.064 | <.001 |
|  | COMM | 0.7589 | 0.0815 | 0.599 | 0.919 | 0.605 | 9.307 | <.001 |

## Variances and covariances

Adjusted Model A

|  | | | | 95% Confidence Intervals | |  | | |
| --- | --- | --- | --- | --- | --- | --- | --- | --- |
| Variable 1 | Variable 2 | Estimate | SE | Lower | Upper | β | z | p |
| pcq_11 | pcq_12 | 0.5359 | 0.06228 | 0.4138 | 0.6580 | 0.635 | 8.60 | <.001 |
| pcpis_d16m | pcpis_d17m | 0.0816 | 0.01395 | 0.0542 | 0.1089 | 0.453 | 5.85 | <.001 |
| pcpis_d5m | pcpis_d6m | 0.1036 | 0.01683 | 0.0706 | 0.1366 | 0.336 | 6.16 | <.001 |
| pcq_02 | pcq_04 | -0.2849 | 0.03835 | -0.3600 | -0.2097 | -0.664 | -7.43 | <.001 |
| pcpis_d6m | pcpis_d12m | -0.1155 | 0.01403 | -0.1430 | -0.0880 | -0.418 | -8.23 | <.001 |
| pcpis_d6m | pcpis_d9m | -0.0775 | 0.01416 | -0.1053 | -0.0498 | -0.455 | -5.48 | <.001 |
| pcpis_d5m | pcpis_d7m | 0.0969 | 0.01699 | 0.0636 | 0.1302 | 0.257 | 5.71 | <.001 |
| pcpis_d2m | pcpis_d4m | 0.0558 | 0.01298 | 0.0303 | 0.0812 | 0.273 | 4.30 | <.001 |
| pcpis_d1m | pcpis_d2m | 0.0405 | 0.00966 | 0.0216 | 0.0595 | 0.228 | 4.20 | <.001 |
| pcq_04 | pcq_10 | 0.1979 | 0.04288 | 0.1139 | 0.2820 | 0.255 | 4.62 | <.001 |
| pcq_01 | pcq_04 | -0.1785 | 0.03613 | -0.2493 | -0.1077 | -0.439 | -4.94 | <.001 |
| pcq_01 | pcq_01 | 0.2925 | 0.03636 | 0.2212 | 0.3637 | 0.231 | 8.04 | <.001 |
| pcq_02 | pcq_02 | 0.3265 | 0.03927 | 0.2495 | 0.4034 | 0.204 | 8.31 | <.001 |
| pcq_03 | pcq_03 | 0.6019 | 0.04903 | 0.5058 | 0.6980 | 0.407 | 12.27 | <.001 |
| pcq_04 | pcq_04 | 0.5645 | 0.04946 | 0.4676 | 0.6614 | 0.490 | 11.41 | <.001 |
| pcq_06 | pcq_06 | 0.6240 | 0.05381 | 0.5185 | 0.7294 | 0.399 | 11.59 | <.001 |
| pcq_07 | pcq_07 | 0.7070 | 0.06646 | 0.5768 | 0.8373 | 0.386 | 10.64 | <.001 |
| pcq_08 | pcq_08 | 0.5396 | 0.04993 | 0.4417 | 0.6374 | 0.339 | 10.81 | <.001 |
| pcq_09 | pcq_09 | 0.6499 | 0.07831 | 0.4965 | 0.8034 | 0.401 | 8.30 | <.001 |
| pcq_10 | pcq_10 | 1.0635 | 0.06250 | 0.9410 | 1.1860 | 0.751 | 17.02 | <.001 |
| pcq_11 | pcq_11 | 0.7796 | 0.06163 | 0.6588 | 0.9004 | 0.645 | 12.65 | <.001 |
| pcq_12 | pcq_12 | 0.9126 | 0.07806 | 0.7596 | 1.0656 | 0.703 | 11.69 | <.001 |
| pcq_13 | pcq_13 | 0.2055 | 0.03300 | 0.1408 | 0.2701 | 0.199 | 6.23 | <.001 |
| pcq_14 | pcq_14 | 0.2987 | 0.03932 | 0.2216 | 0.3757 | 0.241 | 7.60 | <.001 |
| pcpis_d1m | pcpis_d1m | 0.2250 | 0.01642 | 0.1928 | 0.2571 | 0.605 | 13.70 | <.001 |
| pcpis_d2m | pcpis_d2m | 0.1400 | 0.01197 | 0.1166 | 0.1635 | 0.468 | 11.70 | <.001 |
| pcpis_d3m | pcpis_d3m | 0.1099 | 0.01098 | 0.0884 | 0.1314 | 0.295 | 10.01 | <.001 |
| pcpis_d4m | pcpis_d4m | 0.2976 | 0.02569 | 0.2473 | 0.3480 | 0.609 | 11.59 | <.001 |
| pcpis_d5m | pcpis_d5m | 0.3780 | 0.02961 | 0.3200 | 0.4361 | 0.611 | 12.77 | <.001 |
| pcpis_d6m | pcpis_d6m | 0.2510 | 0.02059 | 0.2107 | 0.2914 | 0.627 | 12.19 | <.001 |
| pcpis_d7m | pcpis_d7m | 0.3761 | 0.02466 | 0.3278 | 0.4244 | 0.443 | 15.25 | <.001 |
| pcpis_d8m | pcpis_d8m | 0.4371 | 0.03463 | 0.3693 | 0.5050 | 0.495 | 12.62 | <.001 |
| pcpis_d9m | pcpis_d9m | 0.1154 | 0.01438 | 0.0873 | 0.1436 | 0.130 | 8.03 | <.001 |
| pcpis_d10m | pcpis_d10m | 0.2419 | 0.01763 | 0.2074 | 0.2765 | 0.488 | 13.72 | <.001 |
| pcpis_d12m | pcpis_d12m | 0.3047 | 0.02214 | 0.2613 | 0.3481 | 0.306 | 13.76 | <.001 |
| pcpis_d13m | pcpis_d13m | 0.1144 | 0.01473 | 0.0856 | 0.1433 | 0.290 | 7.77 | <.001 |
| pcpis_d14m | pcpis_d14m | 0.1909 | 0.02162 | 0.1485 | 0.2332 | 0.320 | 8.83 | <.001 |
| pcpis_d15m | pcpis_d15m | 0.1237 | 0.01171 | 0.1008 | 0.1467 | 0.373 | 10.57 | <.001 |
| pcpis_d16m | pcpis_d16m | 0.1661 | 0.01995 | 0.1270 | 0.2052 | 0.494 | 8.33 | <.001 |
| pcpis_d17m | pcpis_d17m | 0.1954 | 0.01908 | 0.1580 | 0.2328 | 0.455 | 10.24 | <.001 |
| SAFE | SAFE | 1.0000 | 0.00000 | 1.0000 | 1.0000 | 0.233 |  |  |
| EVRY | EVRY | 1.0000 | 0.00000 | 1.0000 | 1.0000 | 0.252 |  |  |
| COMM | COMM | 1.0000 | 0.00000 | 1.0000 | 1.0000 | 0.602 |  |  |
| PRE | PRE | 1.0000 | 0.00000 | 1.0000 | 1.0000 | 1.000 |  |  |
| ENV | ENV | 1.0000 | 0.00000 | 1.0000 | 1.0000 | 0.557 |  |  |
| PRO | PRO | 1.0000 | 0.00000 | 1.0000 | 1.0000 | 0.283 |  |  |
| PCQ | PCQ | 1.0000 | 0.00000 | 1.0000 | 1.0000 | 0.482 |  |  |

 Adjusted Model B

|  | | | | 95% Confidence Intervals | |  | | |
| --- | --- | --- | --- | --- | --- | --- | --- | --- |
| Variable 1 | Variable 2 | Estimate | SE | Lower | Upper | β | z | p |
| pcq_11 | pcq_12 | 0.537 | 0.0625 | 0.4147 | 0.659 | 0.63585 | 8.60 | <.001 |
| pcq_02 | pcq_04 | -0.212 | 0.0302 | -0.2711 | -0.153 | -0.45683 | -7.01 | <.001 |
| cdi_04 | cdi_03 | 0.247 | 0.0462 | 0.1560 | 0.337 | 0.29594 | 5.33 | <.001 |
| nci_13 | nci_14 | 0.146 | 0.0323 | 0.0822 | 0.209 | 0.27307 | 4.50 | <.001 |
| nci_15 | nci_18 | -0.208 | 0.0366 | -0.2798 | -0.136 | -0.40338 | -5.69 | <.001 |
| cdi_04 | cdi_08 | 0.328 | 0.0712 | 0.1884 | 0.468 | 0.34112 | 4.60 | <.001 |
| cdi_05 | cdi_08 | 0.381 | 0.0700 | 0.2439 | 0.518 | 0.34431 | 5.44 | <.001 |
| cdi_04 | cdi_05 | 0.273 | 0.0600 | 0.1557 | 0.391 | 0.27505 | 4.56 | <.001 |
| pcq_01 | pcq_01 | 0.358 | 0.0382 | 0.2835 | 0.433 | 0.28346 | 9.38 | <.001 |
| pcq_02 | pcq_02 | 0.322 | 0.0393 | 0.2452 | 0.399 | 0.20128 | 8.20 | <.001 |
| pcq_03 | pcq_03 | 0.529 | 0.0426 | 0.4452 | 0.612 | 0.35693 | 12.42 | <.001 |
| pcq_04 | pcq_04 | 0.668 | 0.0444 | 0.5806 | 0.755 | 0.57439 | 15.04 | <.001 |
| pcq_06 | pcq_06 | 0.639 | 0.0544 | 0.5322 | 0.745 | 0.40882 | 11.75 | <.001 |
| pcq_07 | pcq_07 | 0.713 | 0.0663 | 0.5833 | 0.843 | 0.38897 | 10.76 | <.001 |
| pcq_08 | pcq_08 | 0.526 | 0.0488 | 0.4308 | 0.622 | 0.33126 | 10.79 | <.001 |
| pcq_09 | pcq_09 | 0.646 | 0.0778 | 0.4932 | 0.798 | 0.39884 | 8.30 | <.001 |
| pcq_10 | pcq_10 | 1.052 | 0.0617 | 0.9311 | 1.173 | 0.73068 | 17.04 | <.001 |
| pcq_11 | pcq_11 | 0.781 | 0.0618 | 0.6600 | 0.902 | 0.64656 | 12.65 | <.001 |
| pcq_12 | pcq_12 | 0.913 | 0.0783 | 0.7599 | 1.067 | 0.70328 | 11.66 | <.001 |
| pcq_13 | pcq_13 | 0.211 | 0.0342 | 0.1441 | 0.278 | 0.20443 | 6.17 | <.001 |
| pcq_14 | pcq_14 | 0.292 | 0.0407 | 0.2118 | 0.371 | 0.23550 | 7.16 | <.001 |
| cdi_04 | cdi_04 | 0.863 | 0.0731 | 0.7194 | 1.006 | 0.47649 | 11.80 | <.001 |
| cdi_05 | cdi_05 | 1.144 | 0.0958 | 0.9557 | 1.331 | 0.67335 | 11.93 | <.001 |
| cdi_07 | cdi_07 | 0.827 | 0.0772 | 0.6754 | 0.978 | 0.33313 | 10.70 | <.001 |
| cdi_08 | cdi_08 | 1.072 | 0.0936 | 0.8882 | 1.255 | 0.50906 | 11.45 | <.001 |
| cdi_09 | cdi_09 | 0.644 | 0.0717 | 0.5039 | 0.785 | 0.25337 | 8.98 | <.001 |
| cdi_10 | cdi_10 | 1.121 | 0.1152 | 0.8951 | 1.347 | 0.45502 | 9.73 | <.001 |
| cdi_01 | cdi_01 | 0.893 | 0.1134 | 0.6710 | 1.115 | 0.32793 | 7.88 | <.001 |
| cdi_02 | cdi_02 | 0.393 | 0.0702 | 0.2555 | 0.531 | 0.15421 | 5.60 | <.001 |
| cdi_03 | cdi_03 | 0.805 | 0.0719 | 0.6638 | 0.946 | 0.41322 | 11.20 | <.001 |
| nci_10 | nci_10 | 0.367 | 0.0346 | 0.2995 | 0.435 | 0.36658 | 10.62 | <.001 |
| nci_11 | nci_11 | 0.299 | 0.0320 | 0.2367 | 0.362 | 0.32411 | 9.36 | <.001 |
| nci_12 | nci_12 | 0.272 | 0.0271 | 0.2186 | 0.325 | 0.36591 | 10.01 | <.001 |
| nci_13 | nci_13 | 0.573 | 0.0423 | 0.4901 | 0.656 | 0.52615 | 13.56 | <.001 |
| nci_14 | nci_14 | 0.496 | 0.0383 | 0.4205 | 0.571 | 0.52625 | 12.93 | <.001 |
| nci_15 | nci_15 | 0.492 | 0.0440 | 0.4061 | 0.579 | 0.46109 | 11.18 | <.001 |
| nci_17 | nci_17 | 0.468 | 0.0377 | 0.3943 | 0.542 | 0.39767 | 12.42 | <.001 |
| nci_18 | nci_18 | 0.541 | 0.0579 | 0.4272 | 0.654 | 0.31979 | 9.34 | <.001 |
| nci_19 | nci_19 | 0.727 | 0.0537 | 0.6221 | 0.833 | 0.65198 | 13.54 | <.001 |
| mds_dist_01 | mds_dist_01 | 1.055 | 0.0700 | 0.9178 | 1.192 | 0.54350 | 15.07 | <.001 |
| mds_dist_02 | mds_dist_02 | 1.107 | 0.0746 | 0.9613 | 1.254 | 0.59863 | 14.84 | <.001 |
| mds_dist_03 | mds_dist_03 | 1.041 | 0.0681 | 0.9075 | 1.174 | 0.60223 | 15.29 | <.001 |
| mds_dist_05 | mds_dist_05 | 1.154 | 0.0786 | 1.0005 | 1.309 | 0.72949 | 14.69 | <.001 |
| mds_dist_06 | mds_dist_06 | 1.281 | 0.0786 | 1.1273 | 1.435 | 0.57210 | 16.31 | <.001 |
| mds_dist_08 | mds_dist_08 | 1.084 | 0.0720 | 0.9427 | 1.225 | 0.55164 | 15.05 | <.001 |
| mds_dist_09 | mds_dist_09 | 1.721 | 0.0976 | 1.5293 | 1.912 | 0.67836 | 17.62 | <.001 |
| mds_dist_10 | mds_dist_10 | 1.136 | 0.0777 | 0.9838 | 1.288 | 0.60675 | 14.62 | <.001 |
| mds_dist_11 | mds_dist_11 | 1.448 | 0.0898 | 1.2720 | 1.624 | 0.61253 | 16.13 | <.001 |
| mds_dist_12 | mds_dist_12 | 1.238 | 0.0814 | 1.0784 | 1.398 | 0.61957 | 15.20 | <.001 |
| nci_07 | nci_07 | 0.175 | 0.0512 | 0.0745 | 0.275 | 0.10228 | 3.41 | <.001 |
| nci_08 | nci_08 | 0.417 | 0.0455 | 0.3279 | 0.506 | 0.26743 | 9.17 | <.001 |
| nci_09 | nci_09 | 0.629 | 0.0649 | 0.5013 | 0.756 | 0.30886 | 9.69 | <.001 |
| SAFE | SAFE | 1.000 | 0.0000 | 1.0000 | 1.000 | 0.17889 |  |  |
| EVRY | EVRY | 1.000 | 0.0000 | 1.0000 | 1.000 | 0.29522 |  |  |
| COMM | COMM | 1.000 | 0.0000 | 1.0000 | 1.000 | 0.63454 |  |  |
| EMODIST | EMODIST | 1.000 | 0.0000 | 1.0000 | 1.000 | 0.00811 |  |  |
| EMOWITH | EMOWITH | 1.000 | 0.0000 | 1.0000 | 1.000 | 0.42853 |  |  |
| PERSSAT | PERSSAT | 1.000 | 0.0000 | 1.0000 | 1.000 | 0.37629 |  |  |
| PROFSAT | PROFSAT | 1.000 | 0.0000 | 1.0000 | 1.000 | 0.16863 |  |  |
| MD | MD | 1.000 | 0.0000 | 1.0000 | 1.000 | 0.78201 |  |  |
| IL | IL | 1.000 | 0.0000 | 1.0000 | 1.000 | 0.42669 |  |  |
| CO | CO | 1.000 | 0.0000 | 1.0000 | 1.000 | 0.51106 |  |  |
| JS | JS | 1.000 | 0.0000 | 1.0000 | 1.000 | 0.23614 |  |  |
| PCQ | PCQ | 1.000 | 0.0000 | 1.0000 | 1.000 | 1.00000 |  |  |

## Indirect effects

Model A

|  |  |  | 95% Confidence Intervals | |  |  |  |
| --- | --- | --- | --- | --- | --- | --- | --- |
| Description | Estimate | SE | Lower | Upper | β | z | p |
| PRE ⇒ PRO ⇒ PCQ | 0.087 | 0.125 | -0.158 | 0.331 | 0.06 | 0.695 | 0.487 |
| PRE ⇒ ENV ⇒ PCQ | 0.713 | 0.103 | 0.51 | 0.916 | 0.495 | 6.893 | < .001 |
| PRE ⇒ ENV ⇒ PRO ⇒ PCQ | -0.005 | 0.008 | -0.019 | 0.01 | -0.003 | -0.594 | 0.552 |
| ENV ⇒ PRO ⇒ PCQ | -0.005 | 0.008 | -0.022 | 0.011 | -0.005 | -0.601 | 0.548 |

*Notes*. SE = Standard error, β = Standardised path coefficient.

Model B

|  |  |  | 95% Confidence Intervals | |  |  |  |
| --- | --- | --- | --- | --- | --- | --- | --- |
| Description | Estimate | SE | Lower | Upper | β | z | p |
| MD ⇒ CO ⇒ IL | 0.079 | 0.026 | 0.029 | 0.129 | 0.058 | 3.085 | 0.002 |
| MD ⇒ JS ⇒ IL | 0.122 | 0.046 | 0.033 | 0.212 | 0.09 | 2.683 | 0.007 |
| MD ⇒ JS ⇒ CO ⇒ IL | 0.032 | 0.013 | 0.006 | 0.059 | 0.024 | 2.411 | 0.016 |
| JS ⇒ CO ⇒ IL | -0.116 | 0.03 | -0.175 | -0.058 | -0.156 | -3.9 | < .001 |
| PCQ ⇒ MD ⇒ IL | 0.065 | 0.036 | -0.005 | 0.135 | 0.043 | 1.833 | 0.067 |
| PCQ ⇒ MD ⇒ CO ⇒ IL | -0.042 | 0.014 | -0.07 | -0.013 | -0.027 | -2.901 | 0.004 |
| PCQ ⇒ MD ⇒ JS ⇒ IL | -0.065 | 0.022 | -0.108 | -0.021 | -0.042 | -2.893 | 0.004 |
| PCQ ⇒ MD ⇒ JS ⇒ CO ⇒ IL | -0.017 | 0.007 | -0.03 | -0.004 | -0.011 | -2.541 | 0.011 |
| PCQ ⇒ JS ⇒ IL | -0.717 | 0.098 | -0.909 | -0.524 | -0.468 | -7.293 | < .001 |
| PCQ ⇒ JS ⇒ CO ⇒ IL | -0.19 | 0.049 | -0.285 | -0.094 | -0.124 | -3.897 | < .001 |

*Notes*. SE = Standard error, β = Standardised path coefficient.

## Residual covariances and correlations

Tables are provided in the supplementary Excel file because of their size.
